# Epigenetic Delay in the Neurodevelopmental Trajectory of DNA Methylation States in Autism Spectrum Disorders

**DOI:** 10.3389/fgene.2019.00907

**Published:** 2019-10-01

**Authors:** Michael J. Corley, Nauru Vargas-Maya, Alina P. S. Pang, Annette Lum-Jones, Dongmei Li, Vedbar Khadka, Razvan Sultana, D. Caroline Blanchard, Alika K. Maunakea

**Affiliations:** ^1^Department of Native Hawaiian Health, John A. Burns School of Medicine, University of Hawaii, Honolulu, HI, United States; ^2^Department of Clinical and Translational Research, University of Rochester Medical Center, Rochester, NY, United States; ^3^Office of Biostatistics & Quantitative Health Sciences, John A. Burns School of Medicine, University of Hawaii, Honolulu, HI, United States; ^4^Bekesy Neurobiology Laboratory, Pacific Biosciences Research Center, University of Hawaii at Manoa, Honolulu, HI, United States

**Keywords:** autism, DNA methylation, neurodevelopment, epigenetics, ASD

## Abstract

Autism spectrum disorders (ASD) are hypothesized to originate *in utero* from perturbations in neural stem cell niche regions of the developing brain. Dynamic epigenetic processes including DNA methylation are integral to coordinating typical brain development. However, the extent and consequences of alterations to DNA methylation states in neural stem cell compartments in ASD are unknown. Here, we report significant DNA methylation defects in the subventricular zone of the lateral ventricles from postmortem brain of 17 autism diagnosed compared to 17 age- and gender-matched typically developing individuals. Both array- and sequencing-based genome-wide methylome analyses independently revealed that these alterations were preferentially targeted to intragenic and bivalently modified chromatin domains of genes predominately involved in neurodevelopment, which associated with aberrant precursor messenger RNA splicing events of ASD-relevant genes. Integrative analysis of our ASD and typically developing postmortem brain methylome datasets with that from fetal brain at different neurodevelopmental stages revealed that the methylation states of differentially methylated loci associated with ASD remarkably resemble the methylation states at earlier time points in fetal brain development. This observation was confirmed using additional methylome datasets from three other brain regions. Altogether, these findings implicate an epigenetic delay in the trajectory of normal DNA methylation states during the course of brain development that may consequently lead to deleterious transcriptomic events in ASD and support the hypothesis of an early developmental origin of ASD.

## Introduction

Profound epigenomic remodeling takes place during development, where decisions of cell function and identity are being determined (Chen and Dent, 2014). In the human brain, cell-type specification occurs predominately during prenatal development originating from specific neural stem cell niches, few of which, like the human brain subventricular zone (SVZ), persist into adulthood (Sanai et al., 2011). During this neurodevelopmental window, dynamic epigenetic processes play an important role in regulating transcriptional plasticity suggested to orchestrate brain growth, synaptic pruning, and hodology (Numata et al., 2012). In particular, the brain DNA methylome undergoes extensive, locus-specific changes over the course of neurodevelopment (Lister et al., 2013; Spiers et al., 2015) at genes that influence key neurological and cognitive functions, including neuronal plasticity (Borrelli et al., 2008; Guo et al., 2011), memory formation and maintenance (Day and Sweatt, 2010), and circadian rhythm (Azzi et al., 2014). Deviations to dynamic epigenetic processes including DNA methylation may alter the course of typical brain development and interact with genetics to explain autism spectrum disorders (ASD).

Predominately affecting male individuals, ASD is a group of neurodevelopmental disorders diagnosed according to varying behavioral symptoms including impaired sociality, altered communication, and stereotyped and repetitive behaviors (Fombonne, 2009). Research has revealed that the etiology of ASD is not explained by only genetic alterations (Abrahams and Geschwind, 2008), prompting the notion that environmental and epigenetic factors interact with the genome to alter the course of typical brain development and may contribute to the etiology of idiopathic cases (Loke et al., 2015). Indeed, recent studies have revealed perturbations to epigenetic processes, in particular DNA methylation, in postmortem brain tissue of individuals with ASD compared to typically developing controls (Nagarajan et al., 2006; Ladd-Acosta et al., 2014; Nardone et al., 2014; Zhubi et al., 2014; Wong et al., 2019a). However, little is known about the extent and related molecular consequences of epigenetic perturbations in neural stem cell compartments in the brain of individuals with ASD.

To determine whether epigenetic perturbations occur in a neural stem cell compartment, we focused our analyses on the SVZ—the largest neurogenic niche in the mammalian brain consisting of neural stem cells that proliferate, differentiate, and migrate to form the neocortex during prenatal human brain development (Alvarez-Buylla and Lim, 2004; Sanai et al., 2011). This unique neurogenic region is implicated to contribute to the pathology of ASD presumably through the control of cellular proliferation, apoptosis, and/or cellular migration (Courchesne et al., 2001; Sanai et al., 2004; McCaffery and Deutsch, 2005; Wegiel et al., 2010). Our comparative and integrative analyses of the DNA methylome in 34 postmortem SVZ tissue specimens from ASD cases and age-matched, typically developing individuals reveal new insights into genomic regions targeted for epigenetic disruption and implicates an epigenetic delay in the trajectory of normal DNA methylation states during the course of brain development that may occur in ASD.

## Results

### Immunofluorescence and Epigenetic Confirmation of the Glial Cell Composition of the Human SVZ From Autism-Diagnosed and Typically Developing Individuals

**Figure 1:**
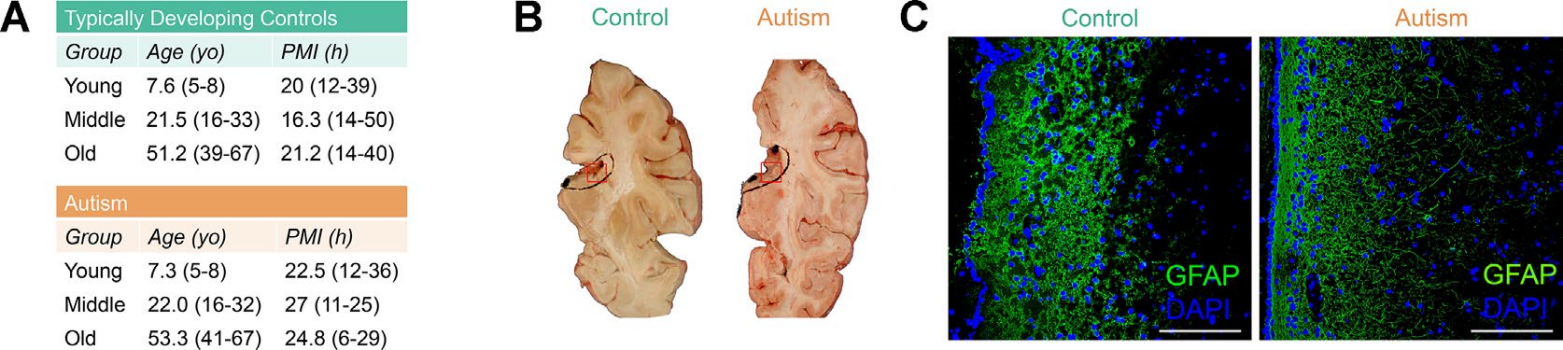
Postmortem subventricular zone (SVZ) tissue from autism-diagnosed and typically developing individuals. **(A)** Postmortem tissue characteristics. yo: years old; PMI: postmortem interval. **(B)** Representative identification of subventricular zone region of interest from fresh frozen postmortem tissue. Red box indicates regions used for analyses. **(C)** Immunofluorescence micrograph displaying layered organization of SVZ region. Glial fibrillary acidic protein (GFAP) (green color) and 4′,6-diamidino-2-phenylindole (DAPI) (blue color) labeling. Scale bar: 100 μm.

Neuropathological abnormalities in the SVZ have been observed in ASD cases (Wegiel et al., 2010; Pearson et al., 2013), yet epigenetic defects in this neural stem cell niche in ASD have never before been reported. To limit potential interindividual variability of DNA methylation due to age, gender, or ethnicity, we selected autism-diagnosed cases (*n* = 17) from the Autism Speaks’ Autism Tissue Program database matched to typically developing controls (*n* = 17) for postmortem interval, gender (all male), and age separated into three distinct groups: “young,” mean age, 7.3; “middle,” mean age, 22.0; and “old,” mean age, 53.3 (**Supplementary Table S1**, **Figure 1A**). No significant differences in age or postmortem interval within these groups were observed (**Supplementary Table S2**). We examined the cellular structure of the SVZ by histology and immunohistochemistry (**Figures 1B, C**) and confirmed cell-type composition by assessing DNA methylation states at a single nucleotide resolution using the Infinium HumanMethylation450 BeadChip (hereafter referred to as the 450k array; **Supplementary Figure S1**). These results agree with previous reports of the cellular composition of the human SVZ harboring glia (Sanai et al., 2004), including astrocyte-like neural progenitors, as the major cell type, and suggest that our data largely reflects this relative homogeneity.

### Aberrant DNA Methylation in the SVZ of Autistic Brain Occurs Preferentially Within Genes Involved in Neurodevelopment

**Figure 2:**
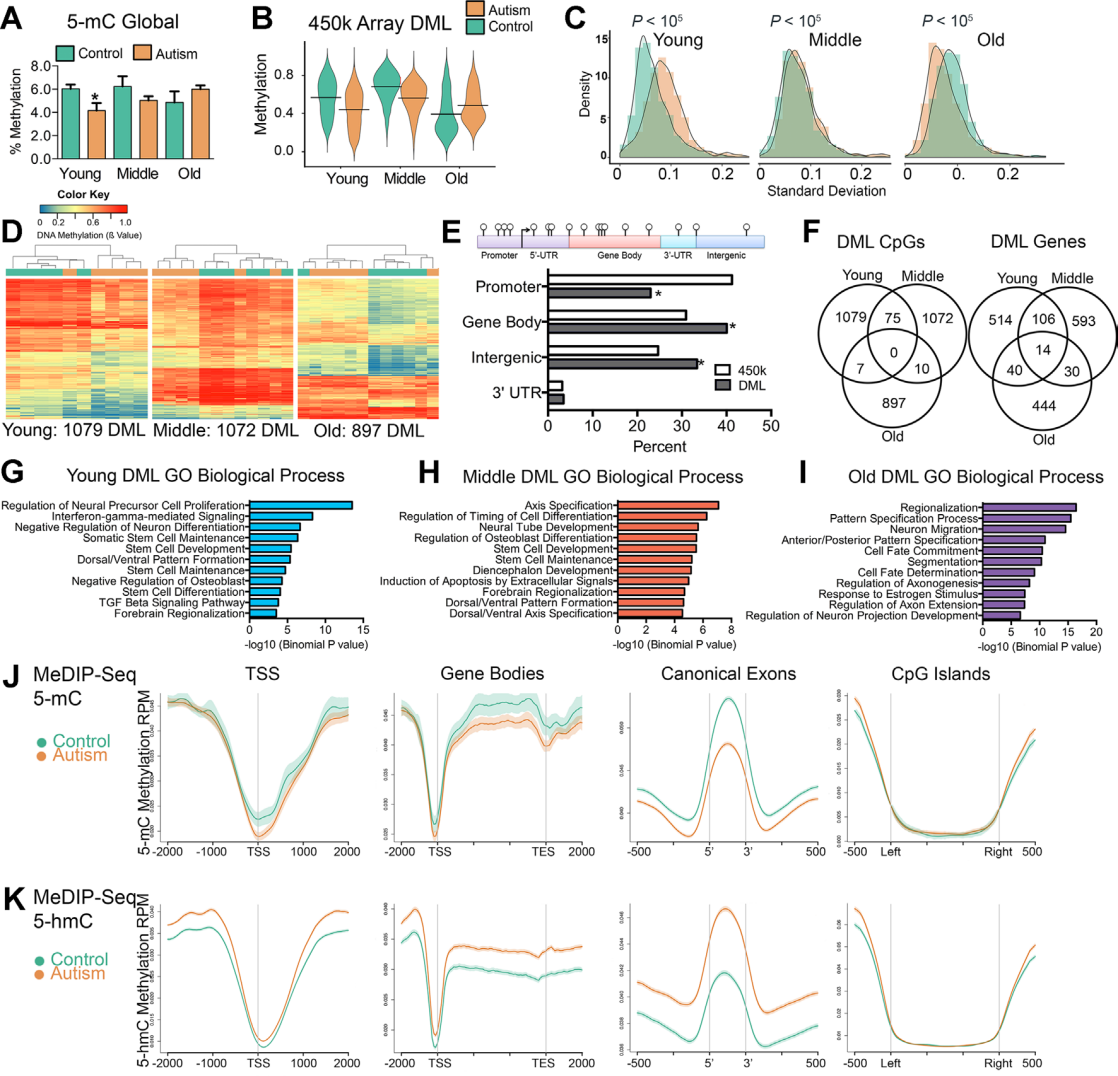
Aberrant DNA methylation in the SVZ region of autistic brain occurs preferentially within genes involved in neurodevelopment. **(A)** Global levels of DNA methylation (5-mC) in control and autism cases. *indicates significant difference between young control and young autism. **(B)** Violin plot of differentially methylated loci between control and autism cases over three age groups. Line represents mean value of percent methylation. **(C)** Density plot comparing the interindividual variability (standard deviation) in levels of DNA methylation for differentially methylated loci (DML) of young, middle, and old typically developing (green) and autism-diagnosed cases (orange). **(D)** Unsupervised hierarchical clustering of the young, middle, and old typically developing (green color) and autism-diagnosed (orange color) cases using a heatmap based on methylation levels of DML. Dendogram shown above. Colors in the heatmap indicate CpG methylation levels (blue to red: low to high methylation levels). **(E)** Percent distribution of all DML in genomic regions compared to distribution of all probes based on the 450k array. *P < 0.05, *χ* ^2^ test. **(F)** Venn diagrams showing the overlap for differentially methylated loci (DML) and DML-related genes for typically developing compared to autism-diagnosed cases at young, middle, and old age groups. **(G–I)** Functional annotation of genes containing differentially methylated loci in autism by database GREAT. Enrichment in gene ontology biological process category shown for all autism-associated DML. **(J)** Distribution of 5-mC methylation and **(K)** 5-hmC methylation over the indicated bp window of all RefSeq annotated TSS, gene bodies, canonical exons, and CpG islands. Methylation of control (green line) and autism (orange line) are displayed as mean RPM values with SEM indicated as a semitransparent shade around the mean curve.

Previous studies reported locus-specific alterations of DNA methylation states in ASD (Nagarajan et al., 2006; Ladd-Acosta et al., 2014; Nardone et al., 2014; Wong et al., 2019b) however, the extent to which this is consistent globally in the SVZ of ASD cases has never been reported. To address this, we measured total 5-methylcytosine (5-mC) content in the bulk DNA from the SVZ of autism-diagnosed and typically developing individuals using an enzyme-linked immunosorbent assay (ELISA). Young-age ASD cases exhibited significant global reductions in DNA methylation compared to typically developing individuals (*P* < 0.05, *t* test), indicating a tendency toward hypomethylation, which was also observed among individuals in the middle age group, albeit nonsignificant (**Figure 2A**). In contrast, older ASD individuals exhibited a slight, nonsignificant increase (**Figure 2A**).

We next determined genome-wide differences in DNA methylation between autism-diagnosed and typically developing individuals by identifying CpG sites from the 450k array dataset that exhibited significant differences (*P* < 0.05) in the levels of DNA methylation between ASD cases and age-matched controls using a resampling-based empirical Bayes method statistical approach with a defined cutoff parameter (mean delta *β* value of ≥10%) and designated these as differentially methylated loci (DML) (Li et al., 2013). We identified 1,079, 1,072, and 897 DML from young, middle, and old age groups, respectively (**Supplementary Dataset 1**). Consistent with the global measures of DNA methylation (**Figure 2A**), we observed that, in general, the levels of DNA methylation among the DML in young and middle age groups were significantly lower [*P* < 0.0001, Kolmogorov–Smirnov (KS) test] in ASD cases compared to controls (**Figure 2B**), in contrast to a significantly higher (*P* < 0.0001, KS test) level of methylation in ASD cases compared to controls in the old age group (**Figure 2B**). Despite the differences of interindividual variability in DNA methylation levels among ASD cases (**Figure 2C**), the DML determined in each age group for the most part stratified ASD from their typically developing counterparts (**Figure 2D**), indicating that most DML changed in the same direction (i.e., either gain or loss) and to a similar magnitude (i.e., methylation level). These DML were significantly underrepresented at promoter regions (*P* < 0.0001, *χ*^2^), yet significantly overrepresented at gene body and intergenic regions of the genome (*P* < 0.0001, *χ* ^2^); we observed no enrichment at 3′-untranslated regions (**Figure 2E**). Surprisingly, the majority of DML identified by age did not overlap (**Figure 2F**), suggesting that alterations to DNA methylation states in ASD may continue to be undergoing age-related changes. However, by determining the underlying genes that these DML are associated with, we observed some overlap between age groups (**Figure 2F**). These data reveal a core set of genes, including those involved in neurodevelopment such as *CXCR7* (Sánchez-Alcañiz et al., 2011), *HDAC4* (Cho and Cavalli, 2014), *LRRFIPI1* (Gurok et al., 2004), *MCF2L* (Raposo et al., 2015), *PRDM16* (Horn et al., 2011), *PTPRN2* (Willi-Monnerat et al., 2008), *SDK1* (Yamagata et al., 2002), and *TRAPCC9* (Mochida et al., 2009), whose CpG methylation levels are commonly altered in ASD independent of age (**Supplementary Table S3**). We then analyzed the biological processes related to the location of the DML within the context of gene networks using the Genomic Regions Enrichment of Annotations Tool (GREAT) (McLean et al., 2010). This analysis indicated that the top Gene Ontology (GO) associated with autism-related DML observed related to genes involved in neuron proliferation, differentiation, and migration among others (**Figures 2G–I**) relevant to the general activity of the SVZ. The GO analysis of all autism-related DML revealed similar biological processes (**Supplementary Figure S2**). Notably, we identified ASD-related DML at clusters of genes involved in embryonic development including homeobox genes, WNT signaling, and neuron differentiation genes (**Supplementary Figure S3**). We confirmed the GO analysis using the Enrichr (Chen et al., 2013) analysis tool (**Supplementary Figure S4**). Next, we sought to evaluate whether any of the DML we identified were related to autism candidate genes that had previously been identified from genetic studies of autism in the AutDB reference. We identified 91 genes associated with ASD by known mutations harboring DML (**Supplemental Tables S4–S6**). These results suggest that the ASD-related alterations of DNA methylation in the SVZ occur preferentially at genes that are involved in neurodevelopmental processes and that are genetically implicated in ASD (Pinto et al., 2014).

Owing to limitations of the array, we expanded our 450k data by obtaining genome-wide coverage of DNA methylation states in five young ASD cases and five typically developing samples utilizing the MeDIP-Seq method (Corley et al., 2015). These data revealed that 5-mC was depleted at transcription start sites (TSS) and CpG islands in the SVZ from typically developing brain, which appeared targeted to gene bodies and canonical exons (**Figure 2J**). This gene pattern of DNA methylation is consistent with our previous findings for the distribution of genome-wide DNA methylation in human brain (Maunakea et al., 2010; Corley et al., 2015). However, compared to that of typically developing individuals, 5-mC in ASD brain was hypomethylated at TSS, gene bodies, and canonical exons (**Figure 2J**), confirming the overall hypomethylation observed in young ASD cases from the 5-mC ELISA assay (**Figure 2A**) and the 450k array (**Figure 2B**). We sought to examine whether the hypomethylation related to ASD could be linked to increased demethylation of 5-mC. Hence, we used MeDIP-Seq to profile the variant 5-hydroxymethylcytosine (5-hmC) in the same five young ASD cases and five typically developing samples we had examined 5-mC. These data revealed that 5-hmC contrasted the distribution of 5-mC and was increased at TSS, gene bodies, and canonical exons (**Figure 2K**). We also observed that 5-hmC was depleted at TSS and CpG islands in the SVZ from typically developing brain (**Figure 2K**). The altered distribution of the variant 5-hmC methylation in our ASD cases is consistent with previous reports from postmortem analyses of ASD cerebellum and mouse models of ASD (Zhubi et al., 2014; Papale et al., 2015) and suggest active demethylation or unresolved poised sites in the genome associate with ASD. To gain further insight into the consequences of these altered methylation states in ASD, we focused on transcriptional regulation.

### ASD-Related DNA Methylation Differences Associates With Altered Pre-mRNA Splicing

**Figure 3:**
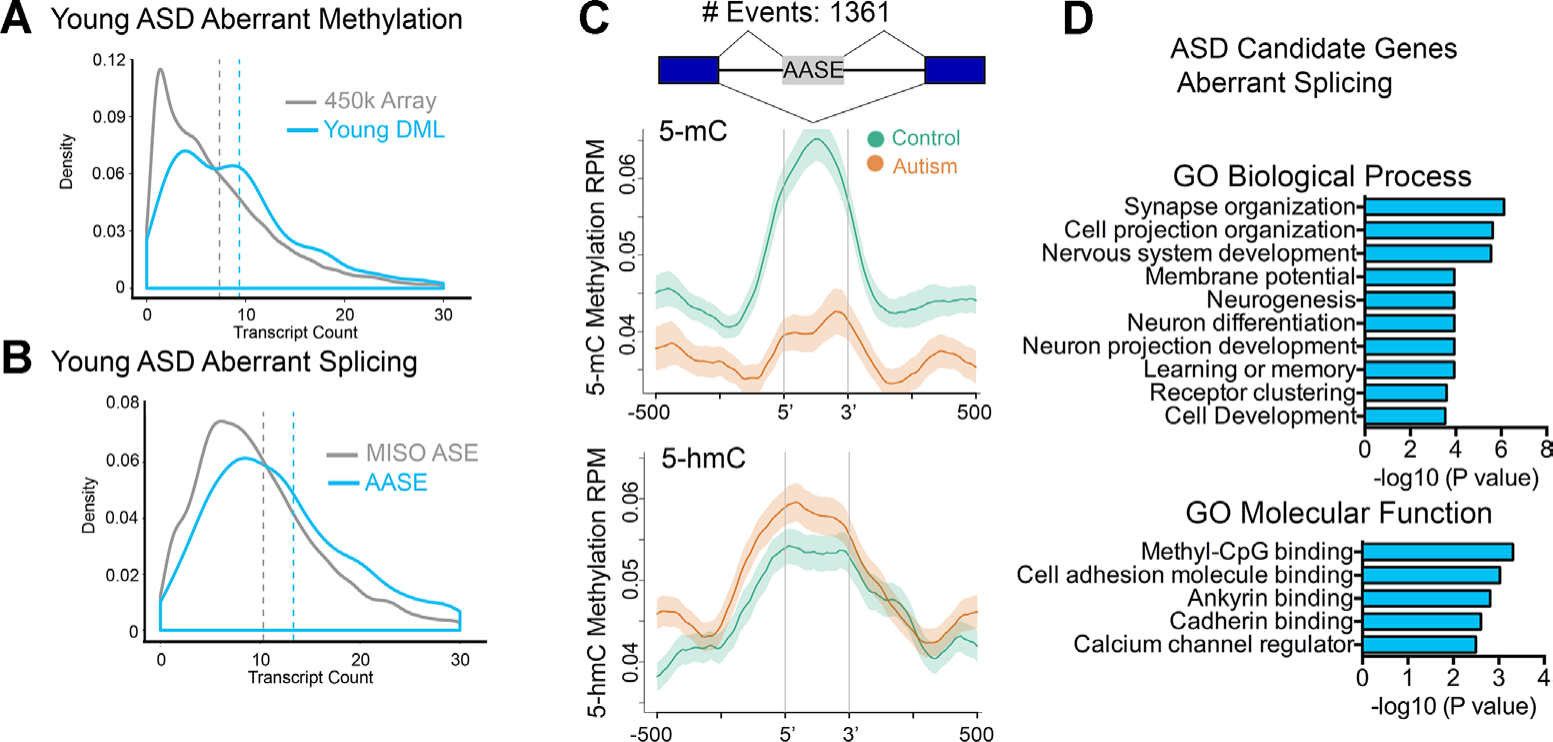
Altered intragenic DNA methylation in ASD associates with aberrant pre-mRNA splicing. **(A)** Density plot of transcript count for gene-associated related CpGs in the 450k array (gray line) and for DML in the young age group (blue line). Dashed lines indicate mean. **(B)** Density plot of transcript count for genes containing alternatively spliced exons annotated in MISO database (gray line) and for aberrantly alternatively spliced exons related to autism in the young age group (blue line). Dashed lines indicate mean. **(C)** Distribution of 5mC and 5hmC methylation over the indicated bp window of all aberrantly alternatively spliced exons (AASEs) associated with autism. Methylation of control (green line) and autism (orange line) are displayed as mean RPM values with SEM indicated as a semitransparent shade around the mean curve. Total number of AASEs is displayed. **(D)** Functional annotation of ASD candidate genes that were aberrantly spliced in the young ASD group by GREAT analysis. Enrichment in Gene Ontology biological process and molecular function categories are shown for young autism group.

Studies from our lab and others have revealed new insights into the relationship of intragenic DNA methylation and transcriptional plasticity, including in tissue-specific alternative promoter usage and precursor messenger RNA (pre-mRNA) splicing (Maunakea et al., 2010; Shukla et al., 2011; Maunakea et al., 2013; Marina et al., 2016) that collectively result in the production of multiple isoforms. Given the preferential distribution of DMLs over intragenic regions in brain relevant genes, we tested the notion that genes harboring multiple isoforms would be altered more frequently than genes harboring fewer isoforms. Indeed, genes whose DNA methylation states were altered in ASD cases tended to be those with significantly more transcript isoforms (e.g., have a higher transcript count) than expected by chance (**Figure 3A**, *P* < 0.0001, KS test). This is consistent with recent reports of ASD-relevant mutagenic events occurring preferentially at longer than shorter genes (King et al., 2013).

That 5-mC hypomethylation occurred precisely over exons and that exon methylation is a determinant of pre-mRNA splicing (Lev Maor et al., 2015) suggested that alternative splicing might be altered in ASD. To test this possibility, we examined gene expression genome-wide using RNA-Seq and employed Mixture of Isoforms (MISO) (Katz et al., 2010) to identify changes in splicing between young ASD and typically developing SVZ cases. We observed extensive alterations in pre-mRNA splicing, mostly of alternatively spliced exons (examples in **Supplementary Figure S5** and **Supplemental Table S7**). Furthermore, aberrant alternative splicing events in ASD tended to occur in genes with a higher transcript count compared to that of all alternatively spliced exons annotated in MISO (**Figure 3B**). To determine whether alterations in DNA methylation associated with aberrant splicing events, we examined all alternatively spliced exons whose levels were altered in ASD cases and evaluated the average MeDIP-Seq inferred methylation states of these exons in young ASD cases compared to that of the age-matched typically developing SVZ specimens. Remarkably, there was a significant depletion of 5-mC over these exons in ASD cases relative to their controls (**Figure 3C**). In comparison, 5-hmC was significantly enriched in ASD cases relative to their controls (**Figure 3C**). GO analysis of aberrantly spliced genes in ASD revealed an enrichment of genes (*P* < 0.001) related to neurodevelopment including synapse organization, cell projection organization, and nervous system development (**Figure 3D**). Interestingly, the methyl-CpG binding protein category was among the top GO molecular function category enriched for ASD candidate genes that we observed to be aberrantly spliced. These findings are consistent with previous postmortem brain studies of autism that report a dysregulation of microexons (Irimia et al., 2014), aberrant alternative splicing of activity-dependent neuron-specific exons (Parikshak et al., 2016), and altered proportion of CPEB4 isoforms resulting from decreased inclusion of a neuron specific microexon (Parras et al., 2018).

### DML Associated With ASD Are Enriched in Bivalent Domains and Enhancers

**Figure 4:**
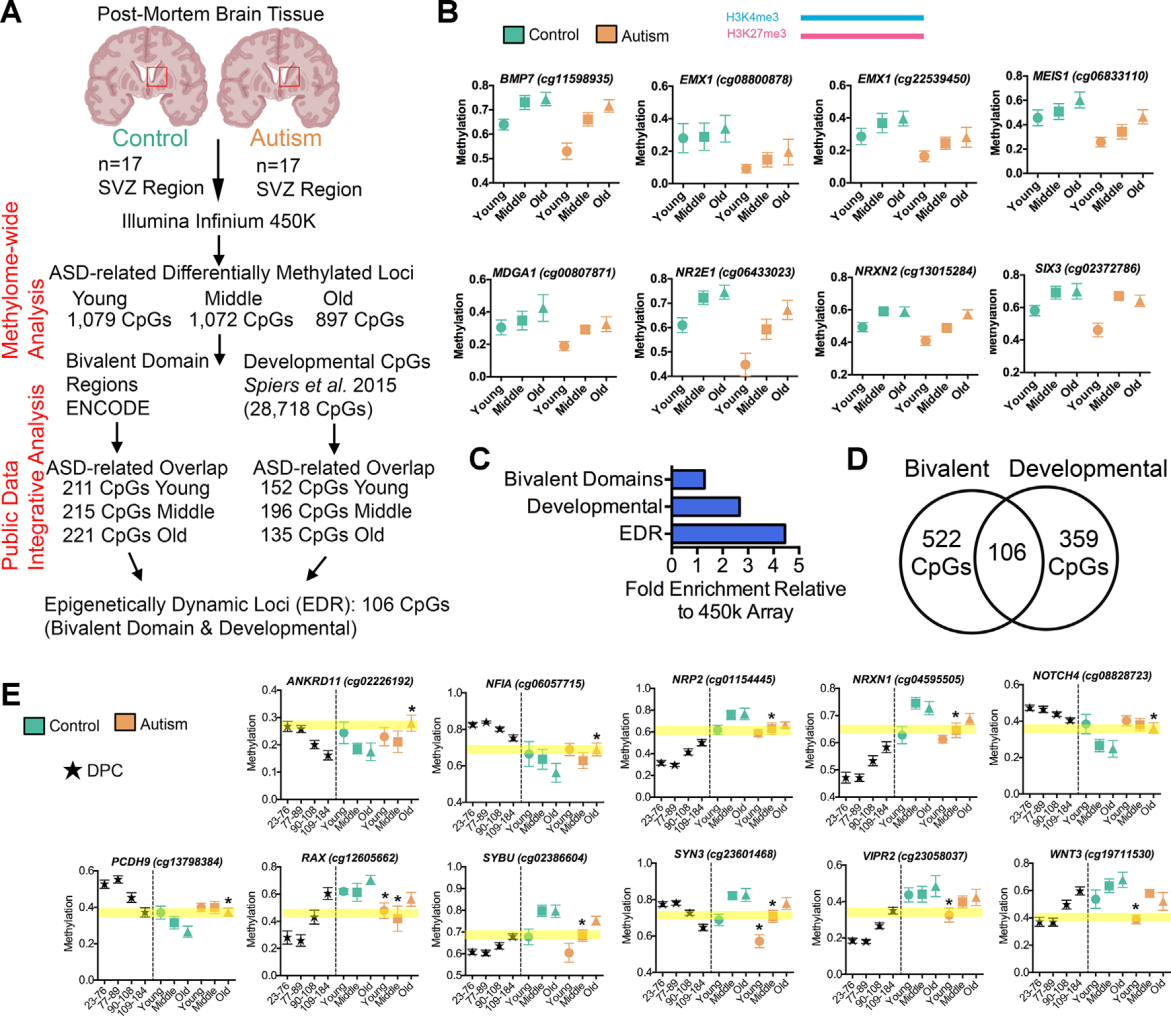
Altered intragenic DNA methylation in ASD occurs in bivalent chromatin domain regions and at CpGs that are developmentally dynamic in fetal brain. **(A)** Flow chart of integrative analysis of 450k array data. **(B)** Autism-associated DML located in a human H1-hESC bivalent domain containing both H3K4me3 (blue bar) and H3K27me3 (pink bar). Typically developing control (green) and autism-diagnosed (orange) mean ± SEM methylation levels presented for DML of related genes by age group. Gene symbol and 450k CpG site indicated above plot. **(C)** Enrichment of autism-associated DML observed in bivalent domains, developmental regions, and EDRs relative to all loci on the 450k array. **(D)** Venn diagram showing the overlap of autism-associated DML that are bivalent domains and developmentally dynamic in fetal brain. **(E)** Autism-associated DML in developmentally regulated regions of the genome. The prenatal human brain days post conception of Spiers et al. (2015) (black stars), typically developing control (green), and autism-diagnosed (orange) mean methylation ± SEM presented for DML of related genes by age group. Gene symbol and 450k CpG probe ID are indicated above graphs. Yellow highlighted region displays the level of methylation that the autism-associated DML is delayed. *, denotes significant methylation difference at P < 0.05 between control and autism samples.

The GO analysis of all DML associated with ASD revealed gene networks involved in neurodevelopment (**Figures 2G–I**), which was independently reinforced by results of the GO analysis of aberrantly spliced genes in ASD (**Figure 3D**). We also observed a general association between DNA methylation and splicing differences between ASD and typically developing individuals (**Figure 3C**). Altogether, these observations suggest a functional, potentially regulatory role for the genomic regions surrounding the DML associated with ASD. To address this, we first determined whether these DML occurred over bivalent chromatin domains, which are defined by the colocalization of activating (i.e., H3K4me3) and repressing (i.e., H3K27me3) histone modifications of chromatin over a genomic region. These domains play a prominent role in genomic imprinting and development and were characterized in embryonic stem cells at promoter regions of genes, poising them for activation (or repression) after resolution during differentiation (Bernstein et al., 2006). We accessed ENCODE H3K4me3 and H3K27me3 ChIP-Seq data on human H1 embryonic stem cells (Consortium, 2012) and identified regions of the genome that contained both marks (20,789 overlapping peaks). We then determined the number of DML overlapping with bivalent domains (observed) and compared this with the frequency of all CpGs on the 450k array that occur within bivalent domains (expected). As indicated in **Figure 4A**, we observed that 211 (19.5%), 215 (20.1%), and 221 (24.6%) of the DML occurred within bivalent domains for the young, middle, and old age groups, respectively. Locus-specific examples of DMLs that overlap with bivalent domains are indicated in **Figure 4B**.

To validate that the DML associated with ASD are enriched in regulatory regions of the genome, we annotated the noncoding regions of the genome containing DML with additional histone modification states from the ROADMAP project for fetal male brain (Skipper et al., 2015). We then examined the ChromHMM chromatin-state annotation consisting of five chromatin marks (H3K4me3, H3K4me1, H3K36me3, H3K27me3, and H3K9me3) for fetal male brain (E081) and observed that a majority (observed: 35.27%, 1,038 CpGs; expected: 19.46%, 573 CpGs, *P* < 0.0001, *χ*^2^) of the ASD-related DML occurred in annotated enhancer regions and enhancer bivalent domains of the genome (**Supplementary Data 1**). These results reinforce the notion that the ASD-related methylation differences occur predominately at regions of the genome with high confidence of *cis*-regulatory activity.

### The DNA Methylation States of the Bivalent Domain-Containing DML in ASD Resemble That of Fetal Brain

Evaluating the developmental timing and extent of epigenetic changes in normal neurodevelopment may offer clues into whether the epigenetic state in ASD deviates from a typical developmental epigenetic trajectory. In particular, the human brain DNA methylome undergoes extensive, locus-specific changes over the course of neurodevelopment at genes that influence key neurological and cognitive functions (Spiers et al., 2017). Deviations to the dynamic trajectory of DNA methylation states may alter the course of typical brain development that later underlies the adverse neurobehavioral consequences observed in ASD. Since the SVZ is critical to early brain development and given that we observed altered ASD-related methylation states are targeted to bivalent domains, we speculated that the aberrant DNA methylation states related to ASD resemble normal methylation states at earlier time points of prenatal neurodevelopment. Such an observation may evidence a form of an “epigenetic delay,” where the methylation states in ASD brain has not been resolved to that of their typically developing counterpart.

**Figure 5:**
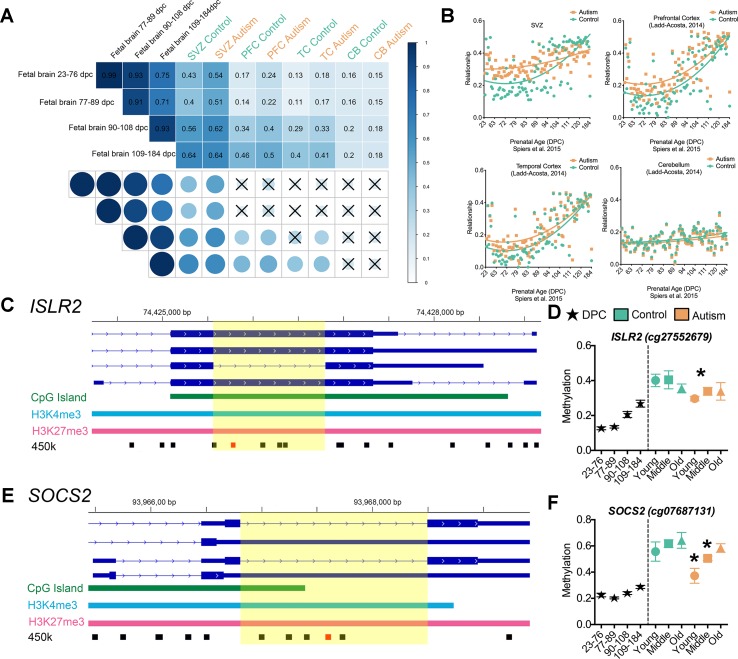
Epigenetic delay of autism-associated DML. **(A)** Relationship between male fetal brain tissue specimens at four stages of development (days postconception, d.p.c.) and postmortem brain tissue from the subventricular zone (SVZ), prefrontal cortex (PFC), temporal cortex (TC), and cerebellum (CB) regions of typically developing (green) and autism-diagnosed (orange) specimens. Correlation matrix showing Pearson correlation coefficient and significance of relationship (*P* < 0.001); *X* denotes nonsignificant. **(B)** Scatterplot of relationship of typically developing control (green) and autism (orange) methylation states at epigenetically dynamic regions versus male fetal brain methylation states from 23 days to 184 d.p.c. for SVZ, PFC, TC, and CB regions. **(C)** Scaled chromosomal view of location of DML in the *ISLR2* gene. CpG island region indicated in green; bivalent domain indicated by H3K4me3 (light blue) and H3K27me3 (pink). Location of all 450k probes indicated in black and location of DML indicated in red. Yellow highlighted region shows DML overlaps with splicing event of exon. **(D)** DNA methylation at one CpG site on the 450k array within the EDR of *ISLR2* progressively increased from low (∼18%) to intermediate (∼40%) levels during fetal brain development and in postnatal SVZ of typically developing control brain. The methylation level at this site in the SVZ from ASD cases remained low (∼30%; ASD young cases), resembling that of fetal brain tissue at 109–184 d.p.c. **(E)** Scaled chromosomal view of location of DML in the *SOCS2* gene. CpG island region indicated in green; bivalent domain indicated by H3K4me3 (light blue) and H3K27me3 (pink). Location of all 450k probes indicated in black and location of DML indicated in red. Yellow highlighted region shows DML overlaps with splicing event of exon. **(F)** DNA methylation level at one CpG site on the 450k array within the EDR of *SOCS2* increased from ∼20 to ∼65% pre- to postnatally in normal brain, while it remained at ∼40–50% in postnatal SVZ of ASD cases. *, denotes significant methylation difference at P < 0.05 between control and autism samples.

To test this hypothesis, we applied an integrative analysis of our 450k methylome data and published 450k data that revealed the trajectory of DNA methylation over the course of human neurodevelopment prenatally (Spiers et al., 2015), where Spiers *et al*. delineated 28,718 CpGs that undergo significant changes across fetal brain development. In our integrative analysis, we examined whether the DML that we identified that associated with ASD may be developmentally regulated in the male fetal brain. We observed, on average, a ∼3-fold enrichment (*P* < 0.001, *χ*^2^) of DML associated with ASD that exhibited normal changes to DNA methylation during fetal brain development than what would have been expected by chance (**Figure 4C**). Locus-specific examples of DMLs that overlap with these developmentally regulated CpGs are indicated in **Figure 4E**. Next, we sought to examine whether these developmentally dynamic DML associated with ASD were enriched in bivalent chromatin domains, which are known to underlie key developmental genes. Interestingly, we found that 106 of the DML associated with ASD overlapped with being located in a bivalent domain and are developmentally dynamic (**Figure 4D**), herein referred to as an epigenetically dynamic region: EDR. A significant fraction (8.9%) of DML associated with ASD occur within EDRs, ∼4.5-fold more frequently than expected by chance (*P* < 0.0001, *χ*^2^; **Figure 4C**). Since the CpGs in ASD-related EDRs both exhibit dynamic methylation states in fetal brain and are localized to known bivalent chromatin domains, we further evaluated their methylation states in the context of neurodevelopment. Specifically, we determined the relationship between the methylation states at each of the 106 CpGs in EDRs in every age group from our ASD cases and typically developing control dataset with that of the methylation states of the same CpGs sites in male fetal brain stratified by prenatal age from the Spiers *et al*. dataset (Spiers et al., 2015) (**Figure 5A**, **Supplemental Table S8**). As expected, the methylation states of CpGs in EDRs from typically developing control SVZ specimens in all age groups exhibited a moderate correlation (*r* = 0.43; *P* < 0.0001) with the earliest prenatal time point for which data was available (23–76 days postconception, d.p.c.); this correlation progressively increased with prenatal age (**Figure 5A**). These data indicate that the methylation states at the EDRs observed in postnatal brain were more similar to late time points in prenatal development (109–184 d.p.c.) than earlier time points. The absolute correlation coefficient at all three age groups were comparable, suggesting that the methylation states at the CpGs driving this association were established early during development and persisted postnatally with age. Intriguingly, we observed that the correlation between the methylation states of the EDRs from ASD cases with that of fetal brain tissue at early prenatal time points (i.e.,23–76 d.p.c., 77–89 d.p.c.) were higher (*r* = 0.54, 0.51) than that of typically developing controls (*r* = 0.43, 0.40) (**Figure 5A**). Next, we sought to expand these findings by accessing 450k data from other brain regions, including the prefrontal cortex (PFC), temporal cortex (TC), and cerebellum (CB) of individuals with autism and typically developing controls (Ladd-Acosta et al., 2014). We then evaluated the relationship between the methylation states of each of the CpGs in EDRs from these three brain regions with that of fetal brain at the different prenatal time points (**Figures 5A, B**). Consistent with our findings from the SVZ, we observed that ASD cases displayed a higher correlation than typically developing controls in PFC and TC (*r* = 0.24, 0.17; *P* < 0.001 and *r* = 0.18, 0.13; *P*< 0.001, respectively). Interestingly, we did not observe such a relationship in CB (*r* = 0.15, 0.16), a region of the brain that the SVZ does not developmentally contribute. These data suggest that the developmental trajectories of DNA methylation states of CpGs in EDRs associated with ASD are specific to the SVZ, PFC, and TC regions.

To exhibit locus-specific examples of this ASD-associated “epigenetic delay,” we characterized the EDRs within the brain-relevant genes immunoglobulin superfamily containing leucine-rich repeat (*ISLR2*) and suppressor of cytokine signaling 2 (*SOCS2*) that interestingly also overlap with alternatively spliced exons (**Figures 5C, E**). The level of DNA methylation at one CpG site on the 450k array within the EDR of *ISLR2* progressively increased from low (∼18%) to intermediate (∼40%) during fetal brain development and in postnatal SVZ of typically developing control brain (**Figure 5D**). Notably, the methylation level at this site in the SVZ from ASD cases remained low (∼30%; ASD young cases), resembling that of fetal brain tissue at 109–184 d.p.c. (**Figure 5D**). Similarly, we observed that the DNA methylation level at one CpG site on the 450k array within the EDR of *SOCS2* increased from ∼20 to ∼65% pre- to postnatally in normal brain, while it remained at ∼40–50% methylation in the postnatal SVZ of ASD cases (**Figure 5F**). It is worth noting that *ISLR2* and *SOCS2* are critically involved in regulating typical axon guidance (Mandai et al., 2009; Mandai et al., 2014) and neuronal differentiation during neural development (Turnley et al., 2002), processes recently shown to be disturbed in the neocortex of children with autism (Stoner et al., 2014). Together, these data suggest that CpGs underlying EDRs in ASD exhibit methylation states reflecting that of early, normal neurodevelopment and acquire the methylation state observed in typically developing control brain later in life.

## Discussion

This study provides the first examination of significant DNA methylation changes in postmortem brain tissue of individuals with ASD focused on a neurogenic region of the brain that is critical to neurodevelopment (i.e., SVZ). Our analysis of postmortem brain specimens from donors at three time points based on age revealed distinct ASD-related DML from young, middle, and old age groups, which occurred preferentially within gene bodies. We observed substantial differences in the methylation levels between ASD and typically developing individuals even after coupling a resampling-based empirical Bayes methods permutation with a stringent cutoff of 10% mean difference, a much more conservative approach than other epigenome-wide DNA methylation studies of postmortem brain (Nagarajan et al., 2006; Ladd-Acosta et al., 2014; Zhubi et al., 2014; Wong et al., 2019a; Nardone et al., 2014). Notably, we observed an ASD-related hypomethylation phenotype in the youngest age group, which was independently observed using three distinct approaches. We identified that the biological processes linked to the ASD-related DNA methylation differences involved genes that play key roles in neurodevelopmental processes and to genes previously implicated in ASD by genetic studies. In addition, we observed that the ASD-related DNA methylation differences occurred at dynamic genomic regions presumed by specific histone modification states to function in transcriptional regulation. Indeed, our integrative analysis of the DNA methylome and transcriptome of the SVZ from young ASD and typically developing individuals revealed an association between intragenic DNA methylation and pre-mRNA splicing abnormalities in ASD. Our findings are consistent with other reports of aberrant DNA methylation in postmortem brain of ASD compared to typically developing controls (Nagarajan et al., 2006; Ladd-Acosta et al., 2014; Nardone et al., 2014; Zhubi et al., 2014; Wong et al., 2019a), and we add to this growing oeuvre genome-wide DNA methylation data from the SVZ, a region of the brain that is critically involved in orchestrating early brain development.

By focusing on the SVZ, we were able to capture a region of the brain consisting mostly of glia cell composition and containing radial glia-like cells and intermediate progenitor cells capable of proliferation (Hansen et al., 2010). Our immunohistochemistry for glial fibrillary acidic protein positive (GFAP+) glia and methylation-based brain cell-type composition analyses confirmed the majority glia cell composition of the SVZ. Previous studies have shown that the frequency of neural stem cells in the SVZ diminishes rapidly after birth and wanes into adulthood (Sanai et al., 2011). In addition, neuropathological data of autism suggest that early in development, autistic brains exhibit aberrant cell growth and neural proliferation, leading to larger brain sizes (Courchesne et al., 2003). Moreover, recent findings using induced pluripotent stem cells from ASD individuals with early brain overgrowth and typically developing controls with normal brain size found that ASD-derived neural progenitor cells displayed increased cell proliferation and ASD-derived neurons displayed abnormal neurogenesis (Marchetto et al., 2017). Our comparative methylomic and transcriptomic analyses of the SVZ independently revealed that genes involved in neuronal differentiation and proliferation were preferentially perturbed in ASD. By comparing the methylation states of CpGs across varying time points in fetal brain development (Spiers et al., 2015), we observed that the developmentally regulated methylation states over functionally relevant loci in ASD we classified as EDRs remarkably appear to resemble that of fetal brain. These results implicate defects to the developmental dynamics of DNA methylation states at early stages of brain development and are not necessarily aberrant events that manifested concurrent with ASD symptoms as suggested by others (Stoner et al., 2014). Altogether, our data indicate epigenetic defects to neural differentiation in the SVZ as a potential source of brain growth abnormalities characteristic of ASD, providing novel molecular support of its early developmental origins. Whether a perturbed DNA methylation signature of neural stem cells residing within the SVZ contributes to ASD-related brain overgrowth remains a compelling question to address in future studies that harness new single cell approaches.

Although our data support the more general observation of genome-wide defects of DNA methylation states in ASD, we acknowledge some limitations of examining postmortem brain tissue specimens that should to be considered. The limited sample size and the clinical heterogeneity of individuals in this study may confound interpretation of our DNA methylation data. The availability of a large number of diagnosed postmortem brains from autistic individuals is difficult to acquire. We chose to focus our analyses by age group to account for this limitation and focus on the brain tissue from young ASD individuals to capture molecular changes that would not be impacted by age-related life-long environmental factors. Moreover, we chose to focus on a neurogenic brain region that is highly relevant to ASD and is comprised of a more homogenous cell type composition albeit mixed. Another limitation is that the resolution of analysis that we performed in postmortem brain for DNA methylation was not at single-cell resolution which would otherwise capture the extent of molecular differences that may be specific to particular subsets of neural and glial cell types, including those that remain ambiguous or uncharacterized. New single-cell bisulfite sequencing methods have emerged that may now permit this level of analysis in future studies (Clark et al., 2017). Despite these limitations, our study revealed exciting new insights into ASD.

Our comparative epigenomic approach using postmortem tissue specimens of ASD cases and age-matched, typically developing controls allowed us to identify specific DMLs associated with ASD. By focusing on DML underlying chromatin states known to be developmentally dynamic (i.e., bivalent domains) and interrogating their methylation states in the context of prenatal brain development, we identified CpGs whose methylation states appeared to be epigenetically delayed. The majority of these CpGs occur at gene body and intergenic regions of the genome with evidence of *cis*-regulatory function, hinting at a potential consequence to gene regulation. Indeed, some of the methylation differences at these CpGs associated with aberrant pre-mRNA splicing that we observed in ASD. Altogether, these findings suggest an epigenetic delay in conditioning the chromatin landscape for transcriptional regulation in neurodevelopment and support pathological observations, implicating the early prenatal origins of ASD. Finally, our study highlights the need to consider molecular analyses on disease-relevant brain regions within a developmental context, which can yield significant implications to understanding the etiology of ASD and other neurodevelopmental diseases despite the technical limitations of postmortem specimens.

## Methods

### Postmortem Brain Tissue Samples

Thirty-four frozen postmortem brain tissue specimens were obtained from National Institute of Child Health Human Development Brain and Tissue Bank for Developmental Disorders (University of Maryland) and Harvard Brain Tissue Resource Center (Boston, MA) through the Autism Tissue Program of Autism Speaks. All cases satisfied criteria for autism based on the autism diagnostic interview revised, the autism diagnostic observation schedule, or were diagnosed by medical records. Typically developing control cases were chosen based on matching age, sex, and postmortem interval. An overview of the samples is provided in **Supplementary Table S1**. To control for anatomical variability, landmarks were chosen and marked with India ink during dissections at each brain bank. Tissue blocks (2.0 cm^3^) were excised from a brain region containing the subventricular zone region of the lateral ventricles and contained portions of the head of the caudate and corpus callosum from fresh frozen coronal sections. A 5-mm tissue punch containing the SVZ region per sample was used for DNA and RNA extractions.

### Nucleic Acid Isolation

DNA and total RNA were isolated from frozen postmortem brain tissue samples using a TissueRuptor (Qiagen Inc) handheld rotor–stator homogenizer and AllPrep DNA/RNA/miRNA Universal kit (Qiagen Inc) according to the manufacturer’s protocol for tissue specimens. DNA/RNA concentration was determined using the Qubit DNA Broad Range or Qubit RNA Broad Range fluorescence assays (Life Technologies) and Qubit Instrument (Life Technologies).

### Immunohistochemistry

Frozen tissue blocks (2.0 cm^3^) were cryosectioned into 10-µm coronal sections and mounted on poly--lysine (1:5 Sigma-Aldrich) coated glass microscope slides (VWR superfrost). Slides were fixed (ice-cold acetone for 3 min), rinsed (2× in phosphate-buffered saline for 5 min), permeabilized (0.5% Triton-X-100 for 15 min), blocked (0.2% phosphate-buffered saline gelatin blocking solution), and immunolabeled using the following antibodies: GFAP anti-GFAP (1:1,000, mouse IgG, Millipore) and Alexa Fluor 488 goat antimouse IgG (1:400, Invitrogen) for 40 min, and covered with a mounting medium containing 4′,6-diamidino-2-phenylindole (DAPI) nuclear marker (Vector Labs, H-1200). Sections were imaged on an Olympus Fluoview FV1000 laser scanning microscope mounted on an Olympus IX81 inverted microscope at the Biological Electron Microscope Facility of the University of Hawaii. DAPI and AlexaFluor 488 were excited sequentially at 405 and 488 nm, respectively, with blue diode and green HeNe lasers. Microscopy images were exported as digital TIFF image files (1,024 × 1,024 pixels).

### Assessment of Global DNA Methylation Levels

Global 5-mC levels in DNA from tissue samples were assessed using a commercial ELISA-based kit (Zymo Research, Irvine, CA, USA). One hundred nanograms of DNA for input samples and standard controls of known methylation were denatured at 98°C for 10 min and coated in triplicate reactions in a 96-well plate. Sample wells were incubated according to manufacturer’s instructions with a primary anti-5-methylcytosine monoclonal antibody specific to 5-mC followed by incubating with an HRP-conjugated secondary antibody (1:1,000 dilution). Next, wells were rinsed, developed with an HRP Developer for 30 min, and the absorbance at 405 nm of each well was read on a VersaMax Microplate Reader (Molecular Devices). Percent 5-mC methylation for each sample was calculated using a logarithmic second-order regression of the standard curve empirically determined from DNA standards of varying DNA methylation, the degree of which was known from manufacturer (Zymo Research).

### Illumina 450k Array-Based DNA Methylation Analyses

Using similar criteria as reported previously (Corley et al., 2015), 500 ng of DNA per sample were bisulfite converted using the EZ DNA Methylation kit (Zymo Research) according to the manufacturer’s instructions. Bisulfite-converted DNA (4 μl per sample) were randomly assigned to a chip well of the Infinium HumanMethylation450 BeadChip to control for potential chip batch effects, amplified, hybridized onto the array, and imaged with the iScan SQ instrument (Illumina) to obtain raw image intensities of methylation for all 34 individual SVZ samples. Array IDAT intensity data were preprocessed in R statistical environment 3.1.2 using the RnBeads 0.99.10 pipeline analysis package (Assenov et al., 2014). Methylation beta values ranging from 0 to 1 (corresponding to unmethylated to methylated signal intensity) for each sample were normalized using the methylumi package and subset-quantile within-array normalization method options. Data were filtered by removing probes containing missing values, SNPs, and/or exhibiting low detection *P* values (detection *P* > 0.05); a total of 18,998 probes were removed after filtering based on these criteria.

#### Cell Composition Analysis

The Cell EpigenoType Specific mapper R package was used to acquire the mean beta values of 10,000 most DML between fluorescence activated cell sorting sorted neurons and glia from male postmortem human brain control tissue samples (Guintivano et al., 2013). Pearson correlation coefficients and *P* values were calculated for the comparisons between the mean beta values of the young, middle, and old control and autism SVZ brain samples to the glia- and neuron-specific DNA methylation states (see **Supplementary Figure S1)**. We found a highly significant association between methylation profiles of both typically developing and autism-diagnosed SVZ specimens to that of glia-specific methylation markers (*r* = 0.93–0.98, *P* < 0.0001). In contrast, we observed a weaker relationship between the neuron-specific methylation profiles of all SVZ specimens to that of neurons (*r* = 0.35–0.42).

#### Differential Methylation

Filtered beta values obtained from the RnBeads pipeline analysis package for all SVZ samples (*n* = 34) were loaded into the R statistical environment 3.1.2. Three planned comparisons of young, middle, and old age groups were analyzed comparing autism and control samples using the resampling-based empirical Bayes methods permutation approach (Li et al., 2013). This approach reduces the false discovery rates for non-normally distributed array-based data and offers higher statistical power. We classified DML as methylation differences >10% (∆*β* value) and *P* < 0.05.

#### Unsupervised Clustering

The DML identified for autism-diagnosed and typically developing samples for each age group comparison were used to generate heatmaps with the gplots R package. Unsupervised hierarchical clustering of the most variable beta values (10% or more change) was calculated using the following clustering method (complete linkage method with Manhattan distance measure).

#### Gene Region Enrichment

The chi-square goodness-of-fit test was used to compare the observed significant differently methylated loci with the expected frequency of CpGs in specific genomic regions (Promoter, Gene Body, Intergenic, 3′-untranslated region) based on the annotation for the complete 450k array.

#### Differentially Methylated Loci-Associated Gene Enrichment Analyses

The HumanMethylation450 manifest file v1.2 was used to obtain genomic position annotations for autism-associated DML. The genomic position and associated HUGO symbol were generated into a BED file. Since the autism-associated DML could either be in coding genes or in noncoding genomic regions, we used the GREAT v.2.0 enrichment tool. BED files were uploaded to the GREAT online tool (http://bejerano.stanford.edu/great/public/html/) and analysis was performed similarly to that previously published (McLean et al., 2010). We also used the DML-related HUGO symbols to perform a gene list enrich analysis with the Enrichr online analysis tool (http://amp.pharm.mssm.edu/Enrichr/)

#### Bivalent Domain Analysis

Human H1-hESC H3K27me3 (Accession: ENCFF001SUY) and H3K4me3 (Accession: ENCFF001SVC) ChIP-Seq data were downloaded from the ENCODE project (Consortium, 2012). We used the BEDTools v.2.21.0 (http://bedtools.readthedocs.org/en/latest/) utility to analyze the processed BED data containing peaks for H3K27me3 and H3K4me3 to identify the overlap between H3K27me3 and H3K4me3. We generated a BED file of the identified H1-hESC bivalent domains. Next, we determined the number of ASD-associated DML (young, middle, and old age groups) and 450k loci located within a bivalent region. Fold enrichment was calculated relative to the calculated percentage of autism-associated DMLs identified in bivalent domains for all loci on the 450k array.

#### Fetal Brain Developmental Trajectory Analyses

450k data from Spiers et al. (2015) was downloaded from the online tool (http://epigenetics.iop.kcl.ac.uk/fetalbrain/). BEDTools v.2.21.0 was used to calculate the number of autism-associated DML that overlapped with the 28,718 CpGs that undergo significant changes over the course of prenatal human neurodevelopment. BEDTools v.2.21.0 was also used to calculate the number of all 450k loci that overlapped with the 28,718 CpGs that undergo significant changes over the course of prenatal human neurodevelopment. Fold enrichment was determined relative to the calculated percentage of autism-associated DML identified in developmentally regulated CpGs for all loci on the 450k array. Methylation data for 100 male fetal brain samples ranging from 23 to 184 days postconception was used to calculate the mean beta values at four defined stages postconception (23–76, 77–89, 90–108, and 109–184) at autism-associated DML from SVZ.

#### Epigenetically Dynamic Region (EDR) Analysis

We used the BEDTools v.2.21.0 utility to define epigenetically dynamic regions (EDRs) by identifying regions that were both developmentally regulated in fetal brain from the dataset of Spiers et al. (2015) dataset and contained a human H1-hESC bivalent domain (H3K27me3 and H3K4me3). To calculate fold enrichment observed in autism-associated DML in EDRs, we first calculated the frequency of EDRs for all loci on the 450k array to obtain the expected frequency. Next, we divided the observed DMLs in EDRs over expected DMLs in EDRs.

#### ASD Candidate Gene Analysis

Genes were selected from the SFARI gene database (https://gene.sfari.org/autdb/Welcome.do) and autism informatics portal (http://autism.mindspec.org/autdb/Welcome.do).

#### Gene Size

We used the biomaRt R package’s ensembl hsapiens dataset to calculate transcript count of gene lists for all genes covered by the 450k array and autism-associated DML.

### MeDIP-Seq

#### Library Construction

MeDIP-Seq was performed as previously reported (Corley et al., 2015). MeDIP-Seq libraries for Ion Torrent semiconductor sequencing on the Ion Torrent PGM or Ion Torrent Proton instruments were generated using a modified protocol consisting of Life Technologies’ Ion Plus Fragment Library kit (Catalog No. 4471252) in combination with a commercially available 5-methylcytosine (MagMeDIP Kit, Catalog No. mc-magme-048) immunoprecipitation kit (Diagenode). A nonspecific mouse IgG was used for an immunoprecipitation reaction as a negative control. For 5-mC and MeDIP-Seq, 1 µg of DNA was sheared to a mean fragment size of 300 bp by sonication with the Covaris M220 Focused-ultrasonicator instrument (Covaris) in a 50-μl microTUBE AFA Fiber Screw-CAP (Covaris). Sonicated DNA was end repaired, purified with Agencourt AMPure XP reagent beads (Beckman Coulter), and ligated with Ion Torrent compatible sequencing adapters following the standard Ion Plus Fragment Library Kit protocol. Adapter-ligated DNA was purified using Agencourt AMPure XP reagent beads and immunoprecipitated using a 5-methylcytosine kit (MagMeDIP) MeDIP kit according to the manufacturer’s protocol. Following 5-mC MeDIP, libraries were size selected from 200–400 bp on a 2% agarose E-gel (Invitrogen) and extracted using a MinElute Gel DNA extraction kit (Qiagen) according to the manufacturer’s instructions. Libraries were then amplified at 18 cycles of PCR (95°C, 15 s; 58°C, 15 s; 70°C, 1 min), purified using MinElute columns (Qiagen), and eluted in 16 μl EB (Qiagen). Libraries were assessed for quality and concentration on an Agilent Bioanalyzer instrument. An aliquot of each library was used to assess enrichment efficiency for control methylated DNA compared to 10% input DNA.

#### Semiconductor Sequencing

MeDIP libraries were templated using the Ion OneTouch 2 System instrument with either the Ion PGM Template OT2 200 kit for the PGM instrument or the Ion PI Template OT2 Kit v2 for the Proton instrument according to manufacturer’s protocols (Life Technologies). Following template reactions, templated libraries were assessed for quality using the Ion Sphere Quality Control Kit (Catalog No. 4468656) and Qubit fluorometer (Life Technologies). Templated libraries that passed manufacturer recommended criteria were enriched using the Ion OneTouch ES instrument. Immediately after enrichment, MeDIP libraries were sequenced on either an Ion PGM or Ion Proton Semiconductor Sequencing machine (Life Technologies).

#### MeDIP-Seq Analysis and Visualization

Ion Torrent sequencing reads were quality-filtered and aligned to the human genome assembly hg19 using Torrent Suite Software’s (v4.0) TMAP alignment software. Alignment files were processed into the Binary Alignment Map (BAM) format and used for further analyses. The ngs.plot software program (Shen et al., 2014) was used to plot and compare the enrichment of methylation over annotated genomic regions (e.g., transcription start sites, gene bodies, CpG islands, and exons). Integrative Genomics Viewer (IGV) (Thorvaldsdottir et al., 2013) was used to visualize MeDIP-Seq areas of enrichment at defined genomic positions.

### RNA-Seq and Alternative Splicing Analysis

#### RNA Extraction and Library Construction

Total mRNA was isolated from brain tissue specimens using the AllPrep DNA/RNA kit (Qiagen), quantified on a Nanodrop-2000 instrument (Thermo Scientific) and evaluated for quality using RNA 6000 Nanochips read on a Bioanalyzer 2100 instrument (Agilent). Two hundred nanograms of total RNA for five autism and five control samples was used to generate barcoded mRNA libraries with the Truseq RNA sample prep kit V2 (Illumina) according to the manufacturer’s protocol. Barcoded RNA-Seq libraries were randomly assigned and pooled two cases per lane, clustered using the cBot Automated Cluster Generation System, and sequenced using a 100-cycle paired end run on an Illumina Genome Analyzer IIx (Illumina Inc., San Diego, CA) instrument. Raw multiplexed reads were deconvoluted by index and grouped by experimental case ID. Sequence quality was evaluated using the FastQC program. Paired-end reads with mean quality score (*Q*) below 20 were filtered out. Sequencing reads were mapped to the Hg19 assembly of the human genome.

#### Alternative Splicing

BAM files of RNA-Seq data from all autism and control samples were merged into two groups, sorted, and indexed separately. MISO software v.0.5.2 (Katz et al., 2010) was employed using General Feature Format (GFF) annotation for hg19 Ensembl genes from UCSC to identify the isoforms/alternative splicing (skipped exon) events. Using the criterion as described by Maunakea et al. (2013), 12,349 included ASEs with inclusion level Ψ ≥ 0.9 and 9,953 excluded with Ψ ≤ 0.1 were identified. Mapped BAM files for control and autism cases were used to determine the number of alternative splicing events and significant splicing differences between control and autism cases. The exons with the inclusion level change ∆Ψ > 0.1 were regarded “aberrantly downregulated” and those with ∆Ψ < -0.1 were regarded as “aberrantly upregulated.” MISO software was also used to generate a Sashimi plot of RNA-Seq data, highlighting exon–exon junctions.

## Data Availability Statement

All datasets used in this study are listed in **Table 1**. The new datasets we generated for this study can be found in the NIH GEO database. GEO Submission (GSE131706) https://www.ncbi.nlm.nih.gov/geo/query/acc.cgi?acc=GSE131706. 

**Table 1:** Overview of datasets.

Dataset	Origin	Description	Platform	Autism subjects, no.	Ref.	Public availability
1	Human Brain Tissue	Neurogenic SVZ niche from ASD-diagnosed and typically developing controls	450 K	17	Novel Data	GSE131706
2	Human Brain Tissue	Temporal cortex, prefrontal cortex, and cerebellum	450 K	19	Ladd-Acosta et al., 2014	GSE53162
3	Human Brain Tissue	179 typically developing human fetal brain samples	450 K	0	Spiers et al., 2015	GSE58885
4	Human Brain Tissue	Fluorescence activated cell sorting sorted neuronal nuclei and glia	450 K	0	Guintivano et al., 2013	GSE41826
5	H1-hESC	H3K4me3 and H3K27me3 ChIP-Seq	ChIP-Seq	0	Bernstein et al., 2006	ENCODE
6	Human Brain Tissue	Neurogenic SVZ niche from ASD-diagnosed and typically developing controls	RNA-Seq	5	Novel Data	GSE131706

## Ethics Statement

The study utilized de-identified postmortem specimens and was reviewed by the University of Hawaii (UH) Human Studies Program. They determined that this study does not qualify as exempt or nonexempt research, and it does not require review and approval by the Human Studies Program or a UH Institutional Review Board (IRB). CHS#2016-30171.

## Author Contributions

MC and AM conceived of and designed the study and wrote the manuscript. NV-M and AP performed MeDIP-Seq and validation experiments. AL-J performed 450k array experiments. DL, RS, and VK analyzed datasets. DB suggested the focus on the SVZ region.

## Funding

This work was supported in part by National Institutes of Health grants P30GM103341, U54MD007584, G12MD007601, U54MD007601, P20GM103457, K01HL125504 (AM), R21MH114005 (AM), P20GM113134, and K01HL140271 (MC) as well as by the Queen’s Health Systems Native Hawaiian Health Initiative, Queen’s Medical Center (MC and AM).

## Conflict of Interest

The authors declare that the research was conducted in the absence of any commercial or financial relationships that could be construed as a potential conflict of interest.
