# Supplementary material for: Epigenetic Delay in the Neurodevelopmental Trajectory of DNA Methylation States in Autism Spectrum Disorders
Source: Front Genet. 2019 Oct 1;10:907. doi: 10.3389/fgene.2019.00907 (PMC6797928; doi:10.3389/fgene.2019.00907)
Supplement: Supplementary file 2 [file Presentation_1.pdf]

Delay in the neurodevelopmental trajectory of DNA methylation associated with autism in post-mortem human brain tissue

SUPPLEMENTARY MATERIALS

Michael J. Corley[1], Nauru Vargas-Maya[1], Alina PS Pang[1], Annette Lum-Jones[1], Dongmei Li[4], Vedbar Khadka[3], Razvan Sultana[1], D. Caroline Blanchard[2], Alika K. Maunakea[1]

[1]Department of Native Hawaiian Health, John A. Burns School of Medicine, University of Hawaii, Honolulu, Hawaii, USA

[2]Bekesy Neurobiology Laboratory, Pacific Biosciences Research Center, University of Hawaii at Manoa, Honolulu, Hawaii, USA.

[3]Office of Biostatistics & Quantitative Health Sciences, John A. Burns School of Medicine, University of Hawaii, Honolulu, Hawaii, USA.

[4]Department of Clinical and Translational Research, University of Rochester Medical Center, Rochester, New York, USA.

Table S1. Clinical characteristics of 34 postmortem subventricular zone (SVZ) tissue samples.

Table S2. Postmortem Brain Tissue Case Characteristics.

Table S3. Overlapping set of genes containing differentially methylated loci among young, middle, and old autism and control groups.

Table S4. Differentially methylated loci for previously identified autism candidate genes in the young age group.

Table S5. Differentially methylated loci for previously identified autism candidate genes in the middle age group.

Table S6. Differentially methylated loci for previously identified autism candidate genes in the old age group.

Table S7. Aberrantly alternatively spliced exons of autism candidate genes.

Table S8. Mean methylation levels (β-values) of autism-associated epigenetically dynamic regions (EDRs) CpG loci

Table S9. Total number of sequenced reads (M, million) from Illumina RNA-Seq libraries aligned to the hg19 assembly of the human genome.

Figure S1. Validation of Glia Cell Composition of SVZ using Cell Type Specific Reference DNA methylation dataset.

Figure S2. Functional annotation of genes containing differentially methylated loci in autism by database GREAT.

Figure S3. Autism-associated differentially methylated loci (DML) for young, middle, and old age groups at homeobox, WNT signaling, and neuron differentiation genes.

Figure S4. Enrichr online gene list enrichment analysis of all autism-associated differentially methylated loci related genes.

Figure S5. Autism-associated differentially methylated loci (DML) for young, middle, and old age groups at homeobox, WNT signaling, and neuron differentiation genes.

Figure S6. Sashimi plots of aberrantly alternatively spliced exons in autism.

Table S1. Clinical characteristics of 34 postmortem subventricular zone (SVZ) tissue samples.

**Autism**				**Typically-Developing Controls**			
Case ID	*Age (y.o)*	*Sex*	*PMI (h)*	*Case ID*	*Age (y.o)*	*Sex*	*PMI (h)*
Young Age Group				***Young Age Group***			
AN08873	5	Male	**25.5**	UMB4332	5	Male	**18**
UMB1349	5	Male	**39**	UMB5408	6.86	Male	**16**
UMB4849	7	Male	**20**	UMB1674	8	Male	**36**
UMB4231	8	Male	**12**	UMB4337	8	Male	**16**
UMB4721	8	Male	**16**	UMB5391	8	Male	**12**
Mean±SEM	*6.6±0.7*		*22.5±4.7*		*7.2±0.6*		*19.6±4.2*
Middle Age Group				***Middle Age Group***			
UMB5403	16	Male	**35**	UMB4669	16	Male	**16**
AN00764	20	Male	**23.7**	UMB1322	16	Male	**25**
UMB4999	20	Male	**14**	UMB1405	21	Male	**13**
AN02639	21	Male	**21.16**	UMB4786	22	Male	**11**
UMB5176	22	Male	**18**	UMB5342	22	Male	**14**
UMB5297	33	Male	**50**	UMB1866	32	Male	**19**
Mean±SEM	*22.0±2.3*		*27±5.4*		*21.5±2.4*		*16.3±2.1*
Old Age Group				***Old Age Group***			
AN06420	39	Male	**13.95**	AN01410	41	Male	**27.17**
AN13287	43	Male	**39.9**	UMB1135	42	Male	**18**
UMB5115	46	Male	**29**	UMB4326	46	Male	**6**
AN09714	60	Male	**26.5**	UMB5498	51	Male	**29**
AN08784	65	Male	**23.13**	AN10723	60	Male	**24.23**
UMB5303	67	Male	**16**	UMB5452	67	Male	**23**
Mean±SEM	*53.33±4.9*		*24.8±3.9*		*51.2±4.3*		*21.2±3.4*

Abbreviations: PMI, postmortem interval; y.o. years old; h, hours; S.E.M. standard error of the mean.

Table S2. Postmortem Brain Tissue Case Characteristics

	Age Test Statistic	PMI Test Statistic
**Young Age (n=5A, 5C)**	$t(8)=0.63$, $p=0.54$	$t(8)=0.46$, $p=0.66$
**Middle Age (n=6A, 6C)**	$t(10)=0.15$, $p=0.88$	$t(10)=1.83$, $p=0.09$
**Old Age (n=6A, 6C)**	$t(10)=0.33$, $p=0.75$	$t(10)=0.68$, $p=0.51$
**Overall (n=17A, 17C)**	$t(34)=0.11$, $p=0.90$	$t(33)=1.84$, $p=0.07$

Abbreviations: A, autism; C, control; PMI, postmortem interval
There were no statistically significant differences between autism-diagnosed cases and typically developing controls for age or PMI.

Table S3. Overlapping set of genes containing differentially methylated loci among young (blue highlight color), middle (red highlight color), and old (purple highlight color) autism-diagnosed and typically developing control groups.

Probe ID	Genomic Position	Gene	Gene Region	p-value	Mean Autism	Mean Control	Beta Diff.
cg21821308	chr2:9533606-9533607	*ASAP2*	Body	0.03	0.49	0.68	-0.19
cg06934572	chr2:9458925-9458926	*ASAP2*	Body	0.01	0.76	0.86	-0.11
cg08844325	chr2:9459368-9459369	*ASAP2*	Body	0.02	0.76	0.91	-0.14
cg09242610	chr2:9357556-9357557	*ASAP2*	Body	0.03	0.40	0.29	0.11
cg17830515	chr7:1151900-1151901	*C7orf50*	Body	0.00	0.75	0.87	-0.12
cg08973950	chr7:1083309-1083310	*C7orf50*	Body	0.01	0.78	0.90	-0.11
cg22785556	chr7:1081061-1081062	*C7orf50*	Body	0.00	0.73	0.91	-0.18
cg20603222	chr7:1096387-1096388	*C7orf50*	Body	0.02	0.78	0.91	-0.13
cg26224354	chr7:1096374-1096375	*C7orf50*	Body	0.02	0.78	0.89	-0.11
cg09423312	chr7:1163549-1163550	*C7orf50*	Body	0.00	0.21	0.11	0.10
cg09462350	chr2:237489273-237489274	*CXCR7*	Body	0.05	0.69	0.83	-0.14
cg15911409	chr2:237481080-237481081	*CXCR7*	5'UTR	0.01	0.46	0.68	-0.22
cg26960322	chr2:237479015-237479016	*CXCR7*	5'UTR	0.04	0.50	0.64	-0.14
cg26960322	chr2:237479015-237479016	*CXCR7*	5'UTR	0.01	0.62	0.76	-0.14
cg03626672	chr2:237478664-237478665	*CXCR7*	5'UTR	0.00	0.29	0.18	0.11
cg00590260	chr10:530805-530806	*DIP2C*	Body	0.02	0.29	0.41	-0.12
cg08324703	chr10:679514-679515	*DIP2C*	Body	0.05	0.47	0.60	-0.13
cg09955645	chr10:531098-531099	*DIP2C*	Body	0.00	0.43	0.53	-0.10
cg26116937	chr10:531152-531153	*DIP2C*	Body	0.01	0.43	0.53	-0.10
cg27421267	chr10:530836-530837	*DIP2C*	Body	0.00	0.52	0.66	-0.14
cg04720116	chr10:679221-679222	*DIP2C*	Body	0.05	0.68	0.80	-0.11
cg08324703	chr10:679514-679515	*DIP2C*	Body	0.04	0.55	0.67	-0.12
cg12014181	chr10:729479-729480	*DIP2C*	Body	0.02	0.44	0.32	0.12
cg12808565	chr10:729259-729260	*DIP2C*	Body	0.01	0.47	0.35	0.12
cg14072016	chr10:384652-384653	*DIP2C*	Body	0.04	0.42	0.30	0.13
cg16936421	chr10:729226-729227	*DIP2C*	Body	0.00	0.51	0.40	0.11
cg10639368	chr2:240241218-240241219	*HDAC4*	Body	0.04	0.56	0.70	-0.14
cg07150777	chr2:239996253-239996254	*HDAC4*	Body	0.04	0.72	0.82	-0.10
cg12877020	chr2:239996263-239996264	*HDAC4*	Body	0.04	0.73	0.84	-0.11
cg25652742	chr2:240152334-240152335	*HDAC4*	Body	0.03	0.48	0.59	-0.11
cg23893620	chr4:20884385-20884386	*KCNIP4*	Body	0.05	0.22	0.33	-0.11
cg06773116	chr4:20884431-20884432	*KCNIP4*	Body, 5'UTR	0.03	0.57	0.68	-0.11
cg27196467	chr4:21305490-21305491	*KCNIP4*	Body, 5'UTR	0.01	0.35	0.24	0.10
cg22121570	chr15:37176940-37176941	*LOC145845*	Body	0.03	0.35	0.50	-0.15
cg26727768	chr15:37178201-37178202	*LOC145845*	Body	0.01	0.44	0.58	-0.15
cg26727768	chr15:37178201-37178202	*LOC145845*	Body	0.03	0.57	0.70	-0.14
cg14475966	chr15:37169665-37169666	*LOC145845*	Body	0.04	0.66	0.77	-0.11
cg04723343	chr2:238647913-238647914	*LRRFIP1*	Body	0.00	0.59	0.70	-0.11
cg04723343	chr2:238647913-238647914	*LRRFIP1*	Body	0.00	0.65	0.76	-0.11
cg15579587	chr2:238600061-238600062	*LRRFIP1*	Body	0.03	0.47	0.31	0.16

cg20732304	chr13:113752657-113752658	*MCF2L*	3' UTR	0.02	0.47	0.60	-0.13
cg17831440	chr13:113699526-113699527	*MCF2L*	Body	0.02	0.54	0.72	-0.17
cg20732304	chr13:113752657-113752658	*MCF2L*	3' UTR	0.05	0.55	0.65	-0.10
cg03309978	chr13:113748255-113748256	*MCF2L*	Body	0.01	0.82	0.92	-0.11
cg03796024	chr10:3151764-3151765	*PFKP*	Body	0.02	0.75	0.92	-0.17
cg20327845	chr10:3138418-3138419	*PFKP*	Body	0.01	0.74	0.84	-0.10
cg20520804	chr10:3149673-3149674	*PFKP*	Body	0.02	0.37	0.25	0.12
cg21401095	chr1:3078839-3078840	*PRDM16*	Body	0.02	0.63	0.82	-0.20
cg22031783	chr1:3128606-3128607	*PRDM16*	Body	0.02	0.50	0.61	-0.12
cg24679453	chr1:3079069-3079070	*PRDM16*	Body	0.00	0.61	0.76	-0.15
cg00806481	chr1:2996650-2996651	*PRDM16*	Body	0.00	0.54	0.66	-0.12
cg09990962	chr1:3164431-3164432	*PRDM16*	Body	0.00	0.62	0.73	-0.10
cg12804677	chr1:3055958-3055959	*PRDM16*	Body	0.01	0.34	0.24	0.10
cg18314778	chr7:157468321-157468322	*PTPRN2*	Body	0.02	0.48	0.61	-0.13
cg20384620	chr7:158122229-158122230	*PTPRN2*	Body	0.02	0.68	0.88	-0.21
cg22517801	chr7:157453790-157453791	*PTPRN2*	Body	0.03	0.76	0.87	-0.11
cg25708755	chr7:157951411-157951412	*PTPRN2*	Body	0.01	0.79	0.92	-0.13
cg08463820	chr7:157453872-157453873	*PTPRN2*	Body	0.02	0.81	0.91	-0.10
cg18008037	chr7:158272029-158272030	*PTPRN2*	Body	0.02	0.67	0.78	-0.11
cg19642306	chr7:157951097-157951098	*PTPRN2*	Body	0.00	0.77	0.90	-0.13
cg22395765	chr7:157362094-157362095	*PTPRN2*	Body	0.03	0.46	0.57	-0.11
cg04207084	chr7:157477846-157477847	*PTPRN2*	Body	0.04	0.28	0.17	0.11
cg05766510	chr7:157454062-157454063	*PTPRN2*	Body	0.01	0.68	0.81	-0.13
cg11575295	chr7:158250911-158250912	*PTPRN2*	Body	0.04	0.57	0.68	-0.11
cg16609021	chr7:157754648-157754649	*PTPRN2*	Body	0.00	0.40	0.53	-0.13
cg17582545	chr7:157647847-157647848	*PTPRN2*	Body	0.00	0.82	0.70	0.12
cg24764310	chr7:157512201-157512202	*PTPRN2*	Body	0.02	0.77	0.90	-0.13
cg27113326	chr7:157454068-157454069	*PTPRN2*	Body	0.02	0.76	0.87	-0.11
cg20943076	chr7:4113480-4113481	*SDK1*	Body	0.00	0.61	0.74	-0.13
cg07249765	chr7:4244643-4244644	*SDK1*	Body	0.01	0.68	0.94	-0.27
cg13818127	chr7:4169630-4169631	*SDK1*	Body	0.04	0.79	0.90	-0.11
cg24441899	chr7:4244372-4244373	*SDK1*	Body	0.00	0.55	0.69	-0.13
cg23357832	chr7:4228775-4228776	*SDK1*	Body	0.02	0.31	0.20	0.11
cg06565641	chr8:141359674-141359675	*TRAPPC9*	Body	0.01	0.65	0.83	-0.18
cg11256152	chr8:141359786-141359787	*TRAPPC9*	Body	0.01	0.65	0.81	-0.16
cg26135849	chr8:141359539-141359540	*TRAPPC9*	Body	0.01	0.51	0.68	-0.17
cg12865888	chr8:141220177-141220178	*TRAPPC9*	Body	0.05	0.61	0.77	-0.17
cg13911501	chr8:141220320-141220321	*TRAPPC9*	Body	0.04	0.59	0.71	-0.12
cg14023573	chr8:140971379-140971380	*TRAPPC9*	Body	0.02	0.77	0.87	-0.10
cg25389145	chr8:141220490-141220491	*TRAPPC9*	Body	0.03	0.56	0.67	-0.11
cg06565641	chr8:141359674-141359675	*TRAPPC9*	Body	0.03	0.76	0.89	-0.12
cg11256152	chr8:141359786-141359787	*TRAPPC9*	Body	0.02	0.75	0.86	-0.11

Table S4. Differentially methylated loci for previously identified autism candidate genes in the young age group. Blue text indicates hypomethylation and red text indicates hypermethylation in autism compared to control.

Probe ID	Genomic Position	Gene	Gene Region	p-value	Mean ASD	Mean Control	Beta Diff.
cg12082025	chr19:1064218-1064219	ABCA7	Body	0.030	0.75	0.90	-0.15
cg04908625	chr3:123166882-123166883	ADCY5	1stExon	0.047	0.32	0.42	-0.11
cg26848724	chr13:31326405-31326406	ALOX5AP	Body	0.008	0.58	0.69	-0.11
cg05320533	chr16:89541815-89541816	ANKRD11	5'UTR	0.023	0.51	0.61	-0.10
cg03452625	chr3:11537620-11537621	ATG7	Body	0.045	0.40	0.51	-0.11
cg23026543	chr7:70203221-70203222	AUTS2	Body	0.023	0.62	0.72	-0.10
cg20446334	chr17:79010458-79010459	BAIAP2	Body	0.014	0.34	0.49	-0.15
cg15867652	chr17:79011277-79011278	BAIAP2	Body	0.046	0.62	0.73	-0.11
cg12363726	chr17:79011542-79011543	BAIAP2	Body	0.013	0.59	0.69	-0.10
cg13610307	chr9:140917431-140917432	CACNA1B	Body	0.020	0.10	0.24	-0.15
cg11827453	chr9:140917413-140917414	CACNA1B	Body	0.027	0.14	0.24	-0.11
cg01799671	chr16:81560752-81560753	CMIP	Body	0.037	0.53	0.64	-0.11
cg01494399	chr16:81709890-81709891	CMIP	Body	0.050	0.64	0.74	-0.10
cg00852921	chr20:62120544-62120545	EEF1A2	Body	0.021	0.50	0.61	-0.10
cg05723219	chr4:187605157-187605158	FAT1	Body	0.023	0.77	0.87	-0.10
cg19062174	chr2:157323390-157323391	GPD2	5'UTR	0.009	0.42	0.55	-0.13
cg14517119	chr5:45678610-45678611	HCN1	Body	0.029	0.74	0.85	-0.11
cg10639368	chr2:240241218-240241219	HDAC4	Body	0.037	0.56	0.70	-0.14
cg24147543	chr6:32554481-32554482	HLA-DRB1	Body	0.008	0.12	0.43	-0.31
cg04601775	chr6:32557478-32557479	HLA-DRB1	1stExon	0.020	0.74	0.63	0.11
cg11993350	chr6:32557404-32557405	HLA-DRB1	Body	0.025	0.79	0.66	0.14
cg00822998	chrX:53310810-53310811	IQSEC2	TSS200;Body	0.029	0.47	0.60	-0.12
cg07787634	chr1:241715378-241715379	KMO	Body	0.028	0.82	0.71	0.12
cg26676468	chr5:112539223-112539224	MCC	Body	0.044	0.43	0.63	-0.20
cg21864959	chr1:11847863-11847864	MTHFR	3'UTR	0.004	0.60	0.70	-0.10
cg09966204	chr13:109741117-109741118	MYO16	Body	0.038	0.63	0.75	-0.12
cg14463068	chr2:1820315-1820316	MYT1L	Body	0.004	0.53	0.64	-0.11
cg06115810	chr1:66797599-66797600	PDE4B	Body;TSS200	0.024	0.33	0.49	-0.16
cg06616051	chrX:153094332-153094333	PDZD4	Body	0.027	0.17	0.30	-0.13
cg00795205	chr16:23916466-23916467	PRKCB	Body	0.020	0.63	0.78	-0.15
cg21464492	chr2:1691062-1691063	PXDN	Body	0.004	0.75	0.92	-0.17
cg04995521	chr1:8468871-8468872	RERE	5'UTR;Body	0.008	0.46	0.59	-0.12
cg20943076	chr7:4113480-4113481	SDK1	Body	0.004	0.61	0.74	-0.13
cg24323958	chr1:108741884-108741885	SLC25A24	Body	0.013	0.52	0.64	-0.12
cg14506366	chr5:1430421-1430422	SLC6A3	Body	0.001	0.71	0.81	-0.11
cg19414711	chr7:127391366-127391367	SND1	Body	0.031	0.61	0.71	-0.10
cg23601468	chr22:33200307-33200308	SYN3	Body	0.026	0.57	0.69	-0.12
cg03709468	chr4:156828285-156828286	TDO2	Body	0.046	0.65	0.76	-0.12
cg03962421	chr7:156943850-156943851	UBE3C	Body	0.021	0.52	0.63	-0.11

Table S5. Differentially methylated loci for previously identified autism candidate genes in the middle age group. Blue text indicates hypomethylation and red text indicates hypermethylation in autism compared to control.

Probe ID	Genomic Position	Gene	Gene Region	p-value	Mean ASD	Mean Control	Beta Diff.
cg05496543	chr16:77467409-77467410	ADAMTS18	Body	0.004	0.70	0.82	-0.12
cg07212541	chr15:101442710-101442711	ALDH1A3	Body	0.004	0.57	0.71	-0.15
cg11099722	chr15:101442813-101442814	ALDH1A3	Body	0.041	0.38	0.51	-0.14
cg02013146	chr16:89452259-89452260	ANKRD11	5'UTR	0.010	0.77	0.91	-0.14
cg03484438	chr16:89452237-89452238	ANKRD11	5'UTR	0.004	0.72	0.87	-0.16
cg14794445	chr15:29396429-29396430	APBA2	Body	0.007	0.55	0.69	-0.14
cg09859040	chr15:80743325-80743326	ARNT2	Body	0.035	0.72	0.82	-0.11
cg03452625	chr3:11537620-11537621	ATG7	Body	0.017	0.58	0.71	-0.13
cg05724997	chr18:60988056-60988057	BCL2	TSS1500	0.012	0.20	0.30	-0.10
cg12090740	chr18:60988058-60988059	BCL2	TSS1500	0.003	0.50	0.64	-0.13
cg10331119	chr1:7209666-7209667	CAMTA1	Body	0.037	0.72	0.84	-0.12
cg24501872	chr1:7562444-7562445	CAMTA1	Body	0.004	0.64	0.75	-0.11
cg27167221	chr1:7103111-7103112	CAMTA1	Body	0.007	0.59	0.69	-0.11
cg05705335	chr16:81520589-81520590	CMIP	Body	0.009	0.76	0.88	-0.11
cg27608563	chr16:81518041-81518042	CMIP	Body	0.008	0.79	0.89	-0.11
cg09269891	chr16:3843415-3843416	CREBBP	Body	0.003	0.64	0.75	-0.11
cg12149795	chr21:47882121-47882122	DIP2A	Body	0.004	0.48	0.58	-0.11
cg03252296	chrX:31283706-31283707	DMD	Body	0.002	0.60	0.71	-0.11
cg21548813	chr6:291882-291883	DUSP22	TSS1500	0.007	0.28	0.53	-0.25
cg20605818	chr10:72166322-72166323	EIF4EBP2	Body	0.005	0.61	0.71	-0.11
cg07110043	chr1:31846397-31846398	FABP3	TSS1500	0.002	0.13	0.43	-0.31
cg08288016	chr4:187590536-187590537	FAT1	Body	0.032	0.78	0.88	-0.10
cg09565404	chr5:108083489-108083490	FER	TSS200	0.010	0.36	0.50	-0.14
cg15225701	chr5:108083455-108083456	FER	TSS200	0.002	0.48	0.68	-0.20
cg07150777	chr2:239996253-239996254	HDAC4	Body	0.039	0.72	0.82	-0.10
cg12877020	chr2:239996263-239996264	HDAC4	Body	0.043	0.73	0.84	-0.11
cg01806261	chr7:110722380-110722381	IMMP2L	Body	0.004	0.58	0.69	-0.11
cg13474918	chr7:119914578-119914579	KCND2	1stExon;5'UTR	0.030	0.71	0.82	-0.11
cg04605816	chr20:62092443-62092444	KCNQ2	Body	0.006	0.69	0.80	-0.11
cg14498592	chr20:62092502-62092503	KCNQ2	Body	0.025	0.42	0.58	-0.16
cg10540110	chr1:202775609-202775610	KDM5B	Body	0.003	0.51	0.62	-0.11
cg01154445	chr2:206548171-206548172	NRP2	Body	0.044	0.63	0.76	-0.13
cg04595505	chr2:50572944-50572945	NRXN1	Body	0.009	0.65	0.75	-0.10
cg13015284	chr11:64410392-64410393	NRXN2	1stExon;Body;5'UTR	0.001	0.49	0.59	-0.10
cg06115810	chr1:66797599-66797600	PDE4B	Body;TSS200	0.005	0.49	0.64	-0.15
cg15070513	chr17:37782685-37782686	PPP1R1B	TSS1500	0.015	0.35	0.45	-0.10
cg00120948	chr1:8484417-8484418	RERE	Body;TSS1500	0.004	0.48	0.61	-0.13
cg19620383	chr15:60860767-60860768	RORA	Body	0.011	0.75	0.86	-0.10
cg23074881	chr15:60872858-60872859	RORA	Body	0.004	0.78	0.88	-0.10
cg05573767	chr6:167171394-167171395	RPS6KA2	Body	0.009	0.41	0.52	-0.11

cg20305005	chr2:166151648-166151649	SCN2A	5'UTR;TSS1500	0.040	0.62	0.73	-0.11
cg20314918	chr2:166151447-166151448	SCN2A	5'UTR;TSS1500	0.034	0.68	0.79	-0.10
cg07249765	chr7:4244643-4244644	SDK1	Body	0.005	0.68	0.94	-0.27
cg13818127	chr7:4169630-4169631	SDK1	Body	0.042	0.79	0.90	-0.11
cg24441899	chr7:4244372-4244373	SDK1	Body	0.003	0.55	0.69	-0.13
cg03520624	chr11:70516558-70516559	SHANK2	Body	0.021	0.70	0.81	-0.11
cg21810373	chr11:70917159-70917160	SHANK2	5'UTR	0.034	0.68	0.81	-0.13
cg02555243	chr12:24097128-24097129	SOX5	5'UTR;Body	0.040	0.66	0.76	-0.10
cg23601468	chr22:33200307-33200308	SYN3	Body	0.008	0.71	0.82	-0.11
cg09228194	chr6:33405457-33405458	SYNGAP1	Body	0.050	0.57	0.68	-0.12
cg01975591	chrX:9432530-9432531	TBL1X	5'UTR;TSS1500	0.047	0.48	0.58	-0.11
cg19506849	chr10:114767609-114767610	TCF7L2	Body	0.001	0.81	0.93	-0.12
cg15637765	chr5:14145681-14145682	TRIO	Body	0.002	0.58	0.69	-0.11
cg09593860	chr11:113929287-113929288	ZBTB16	TSS1500	0.004	0.77	0.87	-0.10
cg14659930	chr3:114128301-114128302	ZBTB20	5'UTR	0.003	0.42	0.54	-0.11

Table S6. Differentially methylated loci for previously identified autism candidate genes in the old age group. Blue text indicates hypomethylation and red text indicates hypermethylation in autism compared to control.

Probe ID	Genomic Position	Gene	Gene Region	p-value	Mean ASD	Mean Control	Beta Diff.
cg10140309	chr20:49548661-49548662	ADNP	TSS1500	0.048	0.58	0.71	-0.14
cg02638523	chr2:236548728-236548729	AGAP1	Body	0.049	0.64	0.53	0.10
cg17665652	chr4:114214093-114214094	ANK2	Body	0.001	0.31	0.21	0.10
cg08311935	chr16:89387805-89387806	ANKRD11	5'UTR	0.002	0.56	0.45	0.11
cg02226192	chr16:89461734-89461735	ANKRD11	5'UTR	0.022	0.28	0.17	0.10
cg15840039	chrX:66788098-66788099	AR	TSS1500;Body	0.032	0.37	0.26	0.10
cg23603995	chr6:157198648-157198649	ARID1B	Body	0.003	0.66	0.37	0.29
cg24825446	chrX:25022499-25022500	ARX	3'UTR	0.049	0.36	0.26	0.11
cg12220696	chrX:77042017-77042018	ATRX	TSS1500	0.002	0.46	0.63	-0.17
cg08992499	chr3:63956514-63956515	ATXN7	Body	0.039	0.53	0.42	0.11
cg19685491	chr12:2800278-2800279	CACNA1C	Body	0.020	0.38	0.24	0.14
cg02794451	chr12:2800446-2800447	CACNA1C	3'UTR	0.045	0.30	0.20	0.10
cg14113970	chr5:110789928-110789929	CAMK4	Body	0.034	0.65	0.53	0.12
cg00647317	chr7:50633725-50633726	DDC	TSS1500	0.015	0.57	0.43	0.13
cg15833940	chr8:1526540-1526541	DLGAP2	Body	0.030	0.53	0.41	0.12
cg00611227	chr2:116122048-116122049	DPP10	Body	0.004	0.70	0.49	0.20
cg04063345	chr6:152130058-152130059	ESR1	Body	0.012	0.41	0.29	0.11
cg15626350	chr6:152130207-152130208	ESR1	Body	0.015	0.27	0.16	0.11
cg24123106	chr6:123100471-123100472	FABP7	TSS200	0.023	0.35	0.25	0.10
cg25652742	chr2:240152334-240152335	HDAC4	Body	0.031	0.48	0.59	-0.11
cg00598125	chr6:32555411-32555412	HLA-DRB1	Body	0.041	0.55	0.33	0.22
cg02361027	chr2:170217401-170217402	LRP2	Body	0.026	0.42	0.29	0.13
cg05180887	chr2:1817263-1817264	MYT1L	Body	0.017	0.69	0.80	-0.11
cg14557268	chr17:29596696-29596697	NF1	Body	0.041	0.45	0.35	0.10
cg06057715	chr1:61554414-61554415	NFIA	Body	0.050	0.69	0.56	0.13
cg07592828	chrX:70364353-70364354	NLGN3	TSS1500	0.009	0.41	0.30	0.11
cg21884374	chr7:107807954-107807955	NRCAM	Body	0.014	0.73	0.63	0.10
cg25622366	chr2:63281139-63281140	OTX1	Body	0.047	0.22	0.10	0.13
cg00160440	chr4:134077575-134077576	PCDH10	Body	0.021	0.37	0.27	0.10
cg13798384	chr13:67636112-67636113	PCDH9	Body	0.008	0.37	0.27	0.11
cg19678392	chr7:94953810-94953811	PON1	1stExon;5'UTR	0.013	0.64	0.49	0.16
cg23591302	chr12:42876090-42876091	PRICKLE1	5'UTR	0.027	0.48	0.31	0.17
cg23357832	chr7:4228775-4228776	SDK1	Body;Body	0.016	0.31	0.20	0.11
cg22335074	chr11:70733258-70733259	SHANK2	Body	0.018	0.49	0.34	0.15
cg11833293	chr11:70557519-70557520	SHANK2	Body	0.015	0.86	0.75	0.11
cg21810373	chr11:70917159-70917160	SHANK2	5'UTR	0.049	0.75	0.86	-0.11
cg16645977	chr7:5342805-5342806	SLC29A4	3'UTR	0.030	0.64	0.76	-0.12

Table S7. Aberrantly alternatively spliced exons of autism candidate genes.

Gene Symbol	Genomic Position	Miso-factor	Delta-Psi	Control Read Count	Autism Read Count	Control Assigned Read Count	Autism Assigned Read Count
ADARB1	chr21:46554630-46554747	2.72	-0.22	14	40	26	58
ANK3	chr10:61841908-61841934	1.00E+12	-0.17	235	158	278	203
APBA2	chr15:29386481-29386516	5.71	-0.12	114	282	179	410
APC	chr5:112074050-112074157	22.09	0.41	25	7	41	13
ATP2B2	chr3:10420927-10420968	4.97	-0.32	16	38	58	155
BIN1	chr2:127825739-127825831	1.00E+12	-0.21	1175	1049	1245	1107
BIN1	chr2:127809831-127809938	63303.83	-0.12	181	276	290	431
CADPS2	chr7:122033250-122033369	2	-0.11	22	50	58	100
CAMSAP2	chr1:200797701-200797733	4.71	-0.17	78	45	106	58
CNR1	chr6:88857383-88857525	23.37	0.12	66	23	2383	767
CSNK1D	chr17:80207574-80208055	35.88	-0.12	129	213	272	369
DEPDC5	chr22:32257354-32257419	2.53	-0.2	27	26	44	57
DLX1	chr2:172951382-172951581	4.65	-0.34	17	25	204	296
DMD	chrX:31144759-31144790	2.68	0.15	40	100	1673	2524
DNAJC19	chr3:180705966-180706017	1225.97	-0.14	119	214	154	273
DNM1L	chr12:32858759-32858797	1.00E+12	-0.32	54	47	133	88
GPHN	chr14:67309396-67309434	8.13	0.16	54	73	54	73
HLA-A	chr6:29912277-29912411	1.00E+12	-0.3	413	1281	1315	3719
HLA-A	chr6:29912277-29912393	1.00E+12	-0.27	760	1831	2389	5220
HMGN1	chr21:40717756-40719218	1.00E+12	0.1	1249	975	1433	1206
HMGN1	chr21:40717756-40717884	147.37	0.26	161	128	338	306
HYDIN	chr16:71061505-71061778	43.62	-0.29	26	74	54	131
HYDIN	chr16:70895951-70896158	2.12	-0.2	23	79	36	116
MBD1	chr18:47801740-47801814	27.14	-0.21	51	41	100	79
MECP2	chrX:153357642-153357765	2.92	0.16	77	97	191	253
NRCAM	chr7:107807366-107807518	1.00E+12	-0.38	114	297	260	608
NRCAM	chr7:107831698-107831727	2272.06	-0.14	152	139	175	163
NRCAM	chr7:107815767-107815802	11.09	-0.12	178	338	465	796
NRCAM	chr7:107866089-107866145	6.17	0.11	72	142	107	215
NRCAM	chr7:107866089-107866145	6.17	0.11	228	266	369	396
NRXN1	chr2:50282093-50282182	54267.35	0.23	149	129	459	435
NRXN2	chr11:64393935-64394024	107740.24	0.12	82	176	404	925
NTNG1	chr1:107961202-107961267	7.67	-0.41	3	47	13	84
NTNG1	chr1:107963706-107963765	13.35	0.22	2	29	6	44
NTRK3	chr15:88524457-88524591	1138448.44	0.19	306	240	3873	3461
PRKD1	chr14:30125682-30125705	291.13	0.28	16	38	58	132
RELN	chr7:103118836-103118841	39.83	0.16	63	71	1604	2050
RGS7	chr1:240963941-240964021	103.42	-0.24	132	238	1171	2052
SBF1	chr22:50895463-50895540	1.00E+12	0.2	73	149	156	317
SETD5	chr3:9476274-9476314	6.24	-0.15	48	39	76	65
SETD5	chr3:9475054-9475246	3.08	0.22	54	35	91	75
SHANK1	chr19:51190274-51190297	15.38	-0.24	37	59	37	59
SLC4A10	chr2:162834232-162834270	1.00E+12	0.3	47	242	731	4440

SNRPN	chr15:25074945-25075052	5.23	-0.28	16	55	46	127
SPAST	chr2:32323865-32323960	2.98	-0.17	39	34	100	83
SYNE1	chr6:152466622-152466690	42.7	0.13	186	258	445	684
SYNGAP1	chr6:33416566-33416645	10.32	-0.11	24	25	3137	5056
SYNGAP1	chr6:33410230-33410271	2.03	0.13	94	165	899	1384
TBC1D5	chr3:17469942-17470011	2.09	-0.13	50	22	63	35
TBC1D5	chr3:17603511-17603634	2.37	0.15	27	31	50	59
TBC1D5	chr3:17627845-17628049	5.96	0.21	27	31	43	53
TTN	chr2:179392173-179392475	2.87	0.28	34	24	180	111
ZNF517	chr8:146029026-146029152	2.32	0.16	45	49	46	53

Table S8. Mean methylation levels (β-values) of autism-associated epigenetically dynamic regions (EDRs) CpG loci for Spiers *et al.'s* typically developing fetal brain, typically developing SVZ, and autism-diagnosed SVZ.

Probe ID	Spiers Fetal 23-76dpc	Spiers Fetal 77-89 dpc	Spiers Fetal 90-108 dpc	Spiers Fetal 109-184 dpc	Young Control SVZ	Young Autism SVZ	Middle Control SVZ	Middle Autism SVZ	Old Control SVZ	Old Autism SVZ
cg00194126	0.20	0.17	0.30	0.45	0.48	0.41	0.66	0.53	0.68	0.57
cg00627029	0.19	0.20	0.26	0.30	0.67	0.57	0.66	0.67	0.47	0.57
cg00766289	0.35	0.33	0.38	0.41	0.61	0.50	0.58	0.46	0.53	0.49
cg01074584	0.45	0.43	0.54	0.67	0.59	0.58	0.75	0.60	0.80	0.71
cg01154445	0.32	0.30	0.41	0.50	0.62	0.59	0.76	0.63	0.77	0.66
cg01270206	0.54	0.53	0.59	0.66	0.68	0.66	0.78	0.68	0.79	0.68
cg01411921	0.22	0.21	0.26	0.33	0.53	0.52	0.69	0.57	0.76	0.71
cg01419914	0.37	0.36	0.48	0.61	0.68	0.62	0.79	0.68	0.78	0.73
cg01692639	0.34	0.34	0.41	0.46	0.47	0.43	0.59	0.49	0.59	0.54
cg01944288	0.40	0.39	0.49	0.58	0.51	0.43	0.63	0.52	0.61	0.55
cg02126031	0.21	0.19	0.23	0.31	0.59	0.54	0.68	0.57	0.71	0.60
cg02362978	0.34	0.35	0.40	0.43	0.44	0.52	0.36	0.38	0.36	0.46
cg02372786	0.18	0.18	0.19	0.22	0.58	0.46	0.69	0.67	0.70	0.64
cg02386604	0.61	0.60	0.64	0.68	0.68	0.60	0.80	0.68	0.80	0.75
cg02387803	0.40	0.34	0.50	0.63	0.62	0.48	0.67	0.55	0.71	0.64
cg02390329	0.58	0.55	0.66	0.71	0.71	0.67	0.87	0.77	0.89	0.87
cg02408252	0.23	0.25	0.28	0.30	0.43	0.44	0.39	0.47	0.39	0.50
cg02821342	0.63	0.61	0.70	0.77	0.44	0.44	0.40	0.29	0.33	0.27
cg02863169	0.46	0.48	0.60	0.68	0.75	0.61	0.86	0.81	0.87	0.80
cg02909446	0.28	0.27	0.38	0.44	0.61	0.60	0.71	0.59	0.73	0.69
cg03599357	0.12	0.11	0.13	0.21	0.42	0.34	0.54	0.40	0.57	0.47
cg04138492	0.30	0.29	0.41	0.52	0.59	0.58	0.71	0.61	0.70	0.61
cg04541368	0.37	0.35	0.47	0.55	0.74	0.62	0.86	0.73	0.89	0.84
cg04595505	0.47	0.47	0.53	0.58	0.63	0.61	0.75	0.65	0.73	0.69
cg04607865	0.18	0.17	0.24	0.27	0.28	0.14	0.36	0.24	0.47	0.21
cg04751149	0.21	0.22	0.27	0.28	0.48	0.42	0.59	0.48	0.60	0.53
cg04797742	0.50	0.50	0.60	0.69	0.73	0.61	0.82	0.79	0.83	0.76
cg05049335	0.36	0.35	0.28	0.23	0.23	0.24	0.29	0.19	0.23	0.16
cg06202276	0.22	0.21	0.25	0.30	0.44	0.34	0.44	0.41	0.48	0.42
cg06814287	0.36	0.36	0.39	0.42	0.40	0.36	0.44	0.32	0.43	0.39
cg07099161	0.23	0.21	0.34	0.45	0.50	0.43	0.56	0.44	0.58	0.49
cg07687131	0.23	0.20	0.24	0.29	0.56	0.37	0.62	0.51	0.64	0.58
cg07833262	0.20	0.20	0.26	0.36	0.51	0.49	0.59	0.48	0.65	0.56
cg07835051	0.25	0.23	0.35	0.48	0.52	0.45	0.56	0.44	0.62	0.51
cg08006625	0.30	0.30	0.36	0.43	0.54	0.50	0.66	0.54	0.64	0.58
cg08239858	0.43	0.43	0.32	0.24	0.17	0.24	0.21	0.32	0.15	0.26
cg08297393	0.55	0.55	0.51	0.43	0.46	0.53	0.36	0.40	0.32	0.45
cg08972081	0.39	0.41	0.42	0.53	0.57	0.50	0.68	0.57	0.66	0.57
cg09012594	0.50	0.52	0.64	0.75	0.66	0.63	0.78	0.67	0.76	0.70
cg09577511	0.33	0.32	0.35	0.38	0.43	0.32	0.45	0.43	0.50	0.41
cg09593860	0.55	0.51	0.70	0.76	0.77	0.68	0.87	0.77	0.79	0.74
cg09626521	0.45	0.44	0.56	0.66	0.67	0.65	0.76	0.62	0.78	0.70

cg09867667	0.25	0.23	0.28	0.34	0.45	0.42	0.56	0.45	0.53	0.53
cg09904497	0.13	0.13	0.14	0.19	0.39	0.35	0.50	0.39	0.50	0.44
cg09941208	0.48	0.47	0.49	0.56	0.76	0.63	0.85	0.77	0.86	0.80
cg09958560	0.55	0.54	0.42	0.34	0.75	0.62	0.87	0.79	0.85	0.84
cg09990962	0.22	0.22	0.27	0.33	0.58	0.55	0.73	0.62	0.74	0.68
cg10201167	0.38	0.39	0.48	0.58	0.49	0.46	0.64	0.52	0.64	0.58
cg10285466	0.51	0.50	0.59	0.66	0.58	0.58	0.72	0.61	0.71	0.67
cg10464312	0.30	0.30	0.40	0.42	0.58	0.53	0.68	0.55	0.67	0.62
cg10502244	0.38	0.39	0.45	0.47	0.40	0.41	0.36	0.44	0.31	0.42
cg10873171	0.60	0.59	0.53	0.43	0.59	0.46	0.69	0.61	0.68	0.62
cg10927841	0.08	0.08	0.13	0.17	0.24	0.13	0.25	0.22	0.34	0.26
cg10941185	0.73	0.72	0.66	0.60	0.68	0.70	0.78	0.68	0.78	0.72
cg10997479	0.23	0.22	0.36	0.50	0.50	0.44	0.56	0.46	0.60	0.49
cg11612799	0.52	0.50	0.57	0.65	0.69	0.62	0.75	0.60	0.80	0.62
cg11692124	0.23	0.24	0.25	0.27	0.56	0.40	0.73	0.58	0.75	0.70
cg12290311	0.55	0.51	0.58	0.65	0.61	0.51	0.75	0.63	0.79	0.75
cg12298745	0.25	0.25	0.29	0.42	0.62	0.55	0.75	0.58	0.75	0.68
cg12427163	0.34	0.36	0.34	0.28	0.43	0.46	0.31	0.42	0.25	0.36
cg12572278	0.18	0.19	0.14	0.13	0.44	0.29	0.36	0.33	0.29	0.30
cg12605662	0.28	0.26	0.43	0.61	0.62	0.48	0.61	0.42	0.70	0.56
cg12732998	0.26	0.24	0.33	0.40	0.51	0.48	0.66	0.56	0.70	0.66
cg13113525	0.12	0.11	0.18	0.25	0.38	0.32	0.48	0.38	0.43	0.39
cg13409439	0.53	0.56	0.50	0.45	0.57	0.50	0.61	0.47	0.54	0.54
cg13474918	0.78	0.78	0.81	0.83	0.77	0.76	0.82	0.71	0.84	0.79
cg13558371	0.49	0.50	0.57	0.63	0.56	0.51	0.61	0.51	0.63	0.60
cg13867255	0.52	0.54	0.46	0.35	0.67	0.63	0.78	0.67	0.68	0.69
cg14031365	0.41	0.39	0.47	0.59	0.52	0.36	0.62	0.49	0.72	0.50
cg14179581	0.17	0.16	0.19	0.23	0.36	0.26	0.38	0.27	0.45	0.28
cg14475966	0.50	0.48	0.58	0.60	0.65	0.58	0.71	0.64	0.77	0.66
cg14573411	0.37	0.35	0.49	0.54	0.52	0.41	0.55	0.41	0.61	0.51
cg14811105	0.27	0.24	0.36	0.50	0.58	0.51	0.73	0.62	0.77	0.67
cg15266508	0.08	0.07	0.11	0.13	0.35	0.24	0.41	0.38	0.39	0.39
cg15329642	0.36	0.35	0.37	0.44	0.62	0.51	0.71	0.63	0.73	0.65
cg15846434	0.19	0.18	0.24	0.30	0.48	0.39	0.63	0.51	0.59	0.54
cg15922174	0.45	0.42	0.58	0.63	0.60	0.50	0.63	0.49	0.70	0.60
cg16134191	0.18	0.18	0.21	0.29	0.43	0.40	0.57	0.45	0.60	0.51
cg16327891	0.52	0.52	0.59	0.65	0.54	0.42	0.64	0.56	0.70	0.57
cg16481280	0.26	0.25	0.29	0.37	0.39	0.35	0.43	0.29	0.40	0.36
cg17178291	0.42	0.42	0.35	0.30	0.35	0.29	0.31	0.36	0.25	0.38
cg17259358	0.30	0.28	0.38	0.41	0.39	0.35	0.54	0.44	0.52	0.45
cg17301216	0.44	0.46	0.37	0.29	0.55	0.50	0.68	0.56	0.72	0.62
cg17310258	0.28	0.29	0.31	0.40	0.58	0.53	0.72	0.61	0.77	0.67
cg18403361	0.39	0.39	0.52	0.62	0.65	0.64	0.82	0.71	0.80	0.77
cg18653451	0.26	0.26	0.23	0.18	0.38	0.27	0.39	0.37	0.41	0.37
cg19630681	0.43	0.45	0.51	0.58	0.58	0.51	0.60	0.56	0.62	0.50
cg20124078	0.28	0.29	0.33	0.36	0.49	0.42	0.60	0.49	0.63	0.52
cg20647118	0.19	0.21	0.22	0.24	0.44	0.39	0.52	0.42	0.52	0.45
cg21639713	0.38	0.37	0.49	0.58	0.60	0.58	0.74	0.64	0.73	0.70

cg22360016	0.32	0.31	0.44	0.45	0.61	0.61	0.71	0.60	0.70	0.65
cg23058037	0.19	0.18	0.27	0.35	0.44	0.33	0.44	0.40	0.48	0.42
cg23662142	0.40	0.37	0.53	0.65	0.74	0.70	0.82	0.76	0.87	0.75
cg23981172	0.54	0.51	0.57	0.64	0.55	0.65	0.64	0.62	0.64	0.63
cg24025550	0.25	0.27	0.29	0.32	0.30	0.18	0.29	0.22	0.38	0.28
cg24036116	0.50	0.53	0.46	0.40	0.32	0.31	0.41	0.30	0.34	0.32
cg24684807	0.39	0.38	0.48	0.54	0.49	0.43	0.63	0.52	0.63	0.58
cg25152404	0.51	0.51	0.56	0.60	0.73	0.67	0.82	0.71	0.82	0.78
cg25615184	0.34	0.35	0.42	0.50	0.44	0.33	0.47	0.43	0.53	0.41
cg25741487	0.23	0.20	0.38	0.59	0.51	0.36	0.60	0.44	0.71	0.50
cg25751787	0.19	0.18	0.24	0.34	0.50	0.47	0.64	0.54	0.65	0.58
cg25900085	0.24	0.24	0.33	0.41	0.53	0.48	0.77	0.64	0.80	0.69
cg26052586	0.52	0.51	0.61	0.65	0.70	0.63	0.76	0.65	0.76	0.69
cg26150922	0.72	0.73	0.68	0.63	0.68	0.76	0.68	0.68	0.62	0.73
cg26218110	0.24	0.22	0.34	0.42	0.60	0.57	0.74	0.63	0.74	0.68
cg27552679	0.13	0.14	0.21	0.27	0.40	0.30	0.40	0.34	0.35	0.34

Table S9. Total number of sequenced reads (M, million) from Illumina RNA-Seq libraries aligned to the hg19 assembly of the human genome.

Sequencer	Sample ID	RIN	Library	Sample Type	Mapped Reads to Human Genome (hg19)
Illumina	AN08873	7.6	RNA-Seq	Autism Human Brain SVZ	46 M
Illumina	UMB1349	7.0	RNA-Seq	Autism Human Brain SVZ	38 M
Illumina	UMB4849	7.3	RNA-Seq	Autism Human Brain SVZ	38 M
Illumina	UMB4231	7.8	RNA-Seq	Autism Human Brain SVZ	37 M
Illumina	UMB4721	7.3	RNA-Seq	Autism Human Brain SVZ	44 M
Illumina	UMB5408	7.0	RNA-Seq	Control Human Brain SVZ	87 M
Illumina	UMB1674	7.4	RNA-Seq	Control Human Brain SVZ	38 M
Illumina	UMB4337	7.3	RNA-Seq	Control Human Brain SVZ	36 M

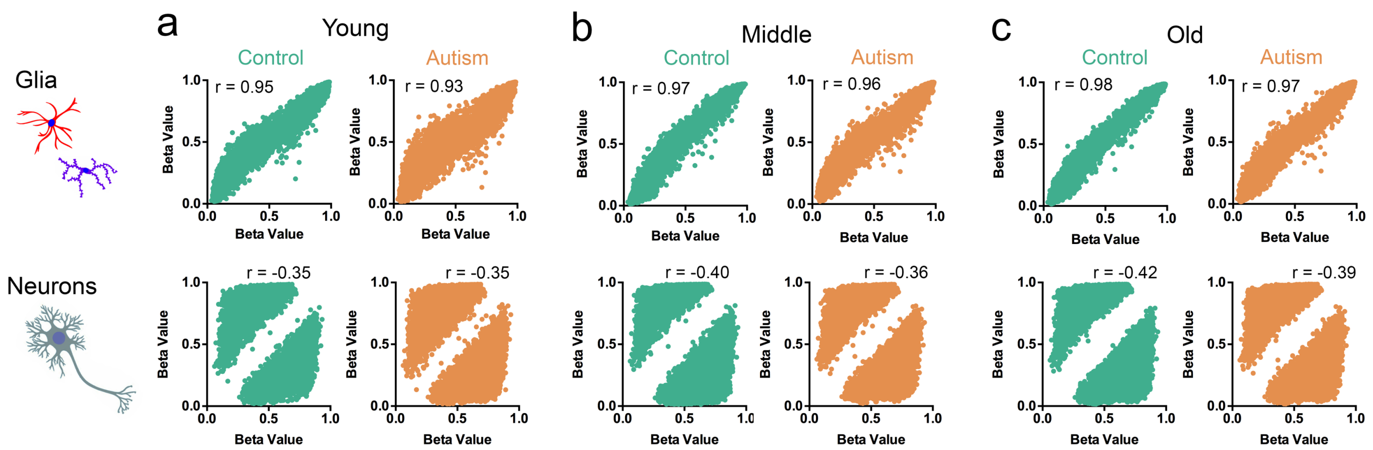

Figure S1. Validation of glia cell composition of SVZ using cell type specific reference DNA methylation datasets for FACS-sorted glia and neurons derived from postmortem human brain tissue. The Cell EpigenoType Specific (CETS) mapper R package was used to acquire the mean beta values of 10,000 most differentially methylated loci between FACS-sorted neurons and glia from postmortem human brain control tissue samples. We found a highly significant association between the glia-specific methylation profiles and that of SVZ specimens of all autism-diagnosed and typically developing individuals. (a)The relationship between glia and neuron-specific methylation markers (y-axis) and young control and autism postmortem brain tissue specimens (x-axis) (b) The relationship between glia and neuron-specific methylation markers (y-axis) and middle control and autism postmortem brain tissue specimens (x-axis) (c) The relationship between glia and neuron-specific methylation markers (y-axis) and old control and autism postmortem brain tissue specimens (x-axis)

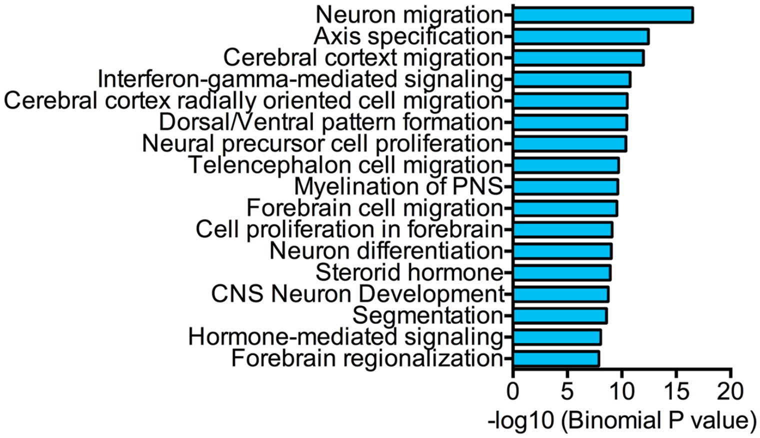

Figure S2. Functional annotation of genes containing differentially methylated loci in autism by database GREAT. Enrichment in gene ontology biological process category shown for all autism-associated DML.

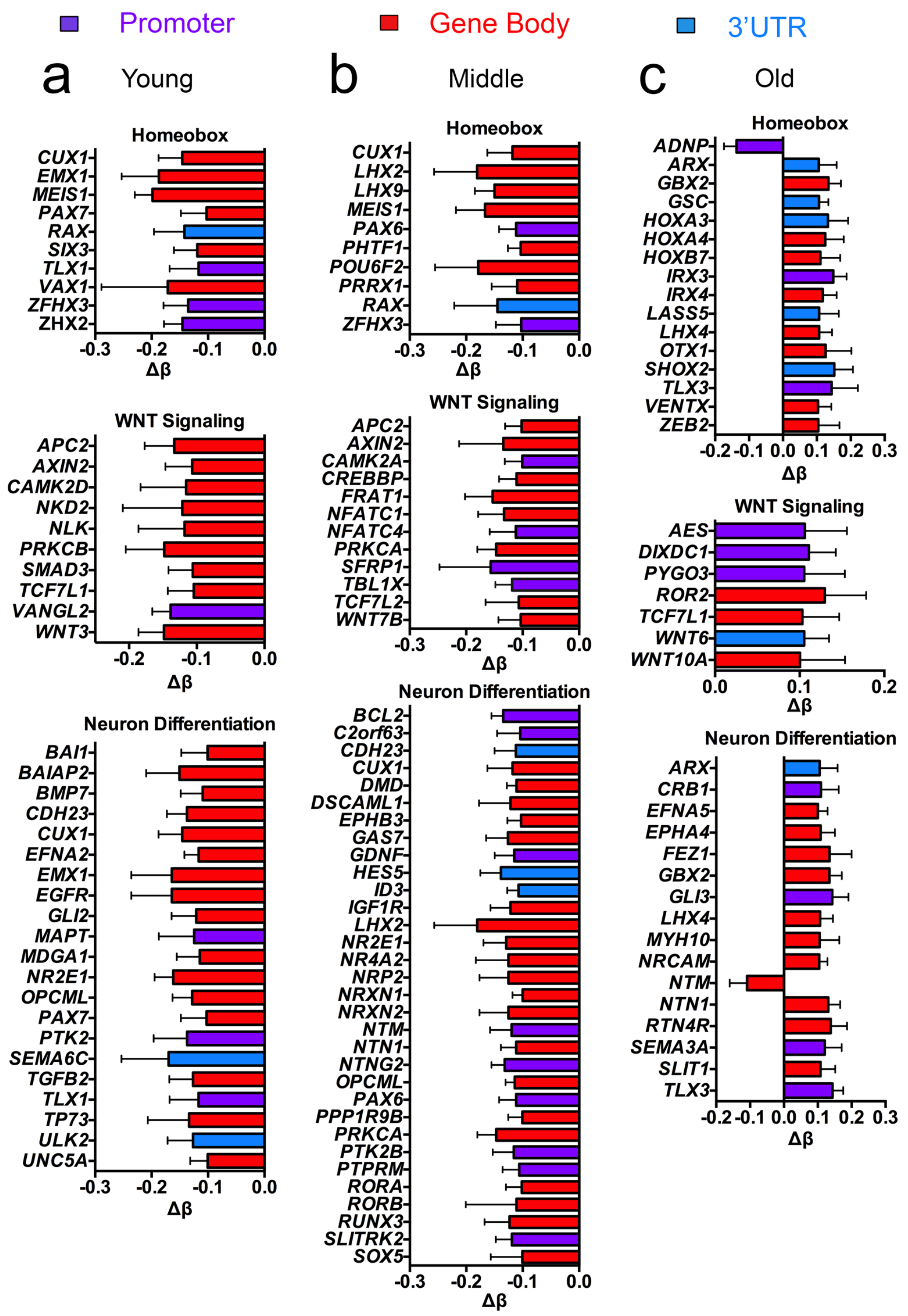

Figure S3. Autism-associated differentially methylated loci (DML) for young, middle, and old age groups at homeobox, WNT signaling, and neuron differentiation genes. DML at promoter (purple), gene body (red), and 3’UTR (blue) regions are presented as the average beta delta difference between autism and typically developing cases.

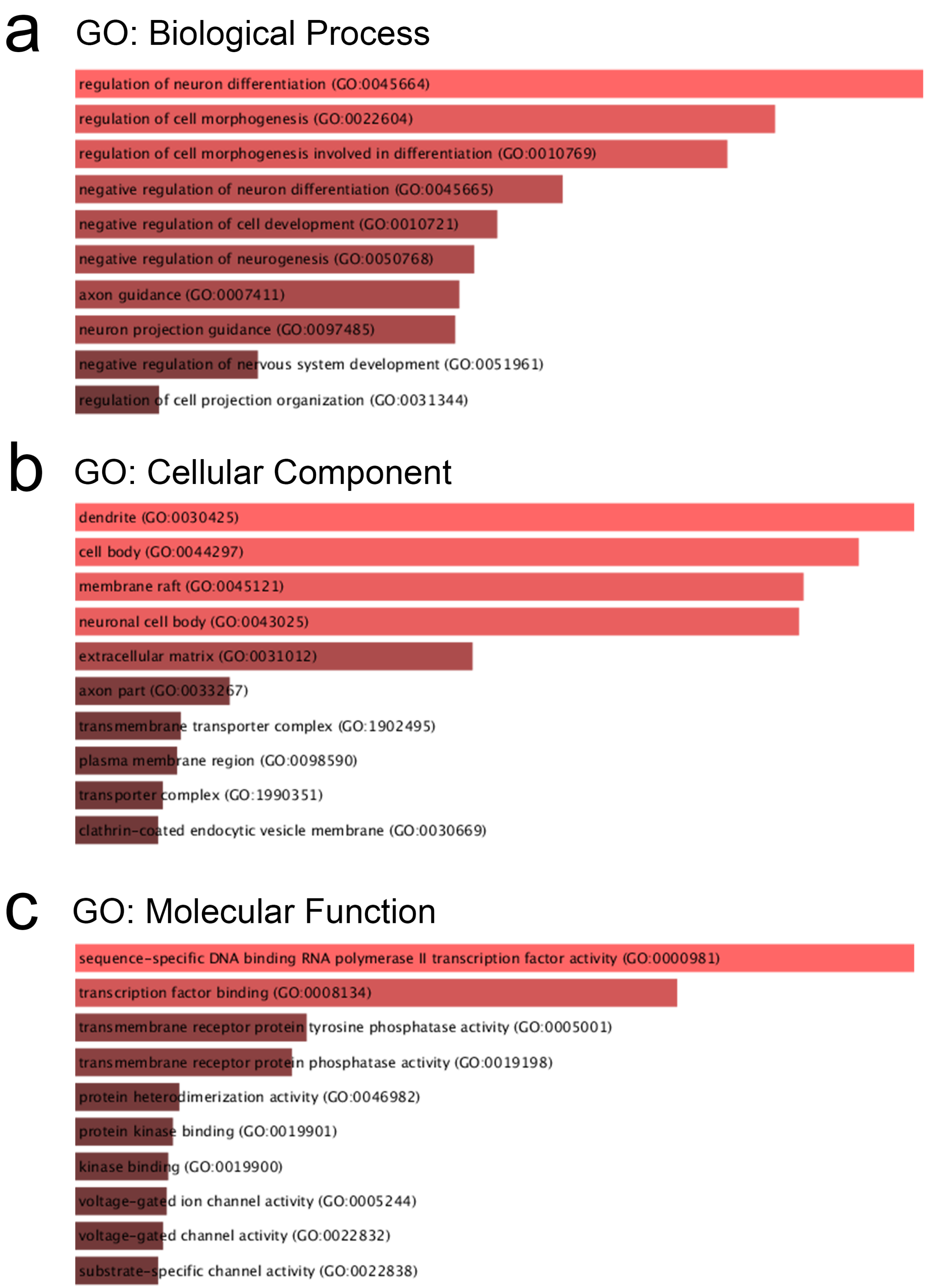

Figure S4. Enrichr online gene list enrichment analysis of autism-associated differentially methylated loci related genes. Enrichment graph of gene ontology (GO) categories a.) biological process, b.) cellular component, and c.) molecular function. Bar graph shows top 10 ranked GO terms sorted by combined score. Combined score calculated using the log of the P-value from the Fisher exact test multiplied by the Z-score of the deviation from the expected rank.

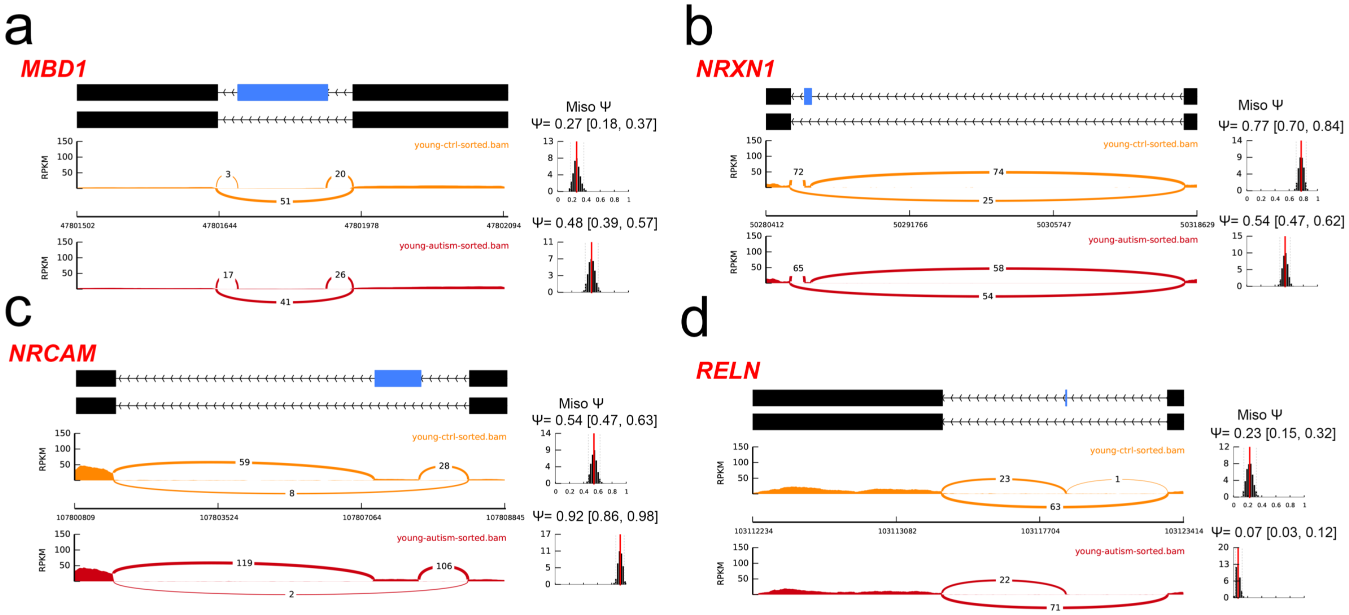

Figure S5. Sashimi plots of aberrantly alternatively spliced exons in autism. Sashimi plot generated by MISO analysis of RNA-Seq data measured as RPKM in control (orange) and autism (red) illustrates the number of exon-exon junction reads as indicated to infer isoform expression. The right graphs show the MISO calculated distribution of a percent exon inclusion score (Psi-value; 95% confidence intervals in brackets) for the (a) *MBD1,* (b) *NRXN1,* (c) *NRCAM,* (d) *RELN* ASEs from control (top) and autism (bottom) RNA-Seq data.
